# Molecular, physiological, and biochemical characterization of extracellular lipase production by *Aspergillus niger* using submerged fermentation

**DOI:** 10.7717/peerj.9425

**Published:** 2020-07-07

**Authors:** Amira Hassan Alabdalall, Norah Ayad ALanazi, Sumayh A. Aldakeel, Sayed AbdulAzeez, J. Francis Borgio

**Affiliations:** 1 Department of Biology, College of Science, Imam Abdulrahman Bin Faisal University, Dammam, Saudia Arabia; 2 Department of Genetic Research, Department of Epidemic Diseases Research, Institute for Research and Medical Consultation (IRMC), Imam Abdulrahman Bin Faisal University, Dammam, Saudia Arabia

**Keywords:** Lipase, *Aspergillus niger*, Submerged fermentation method, Random Amplified Polymorphic DNA, Dendrogram, Unweighted pair group method with arithmetical averages

## Abstract

**Background:**

Extracellular production of fungal lipases especially the lipases obtained from the *Aspergilli* has gained immense interest in recent years due to its diverse biotechnological applications. In this study, we focused on determining the fermentation parameters required for the optimal lipase production.

**Methods:**

A total of 256 fungal isolates were obtained from oil seeds. From each genus, one isolate was selected to evaluate lipase production using phenol red and tributyrin plate assays. Lipase activity was estimated using the spectrophotometric pNPP hydrolysis assay. The highest lipase producer isolates were identified using 18S ribosomal RNA gene sequencing. The genetic variability was determined by random amplified polymorphic DNA (RAPD) analysis and the dendrogram was constructed using the unweighted pair group method with arithmetic averages method. The isolates were examined in a submerged fermentation culture (Smf) to measure the effect of temperature, pH, incubation time, carbon source, nitrogen source, inoculum volume, and lipid source on lipase production.

**Results:**

Eleven isolates belonging to the genus* Aspergillus* were analyzed for lipase production where they were found to be the highest lipase producers among various fungal genera. All the tested isolates were identified as *A. niger* using 18s rRNA sequencing. Genetic diversity was evaluated among all of the studied *A. niger* isolates using RAPD primers. The RAPD primers were used to amplify 285 loci, of which five were polymorphic (1.75%) and seven were monomorphic (2.45%). Thus, a high level of genetic diversity was observed among all isolates. The tributyrin test and the lipase activity assay identified five strains of *A. niger* as high lipase producers, and their optimal enzyme activities were 709.74, 532.54, 735.64, 794.62, and 787.69 U/ml. The optimal conditions for lipase production were as follows: 40 °C, pH 7.5, 1% fructose as the carbon source, 1% yeast extract as the nitrogen source, 2% palm oil, 2.5 × 10^7^ spores/ml suspension, and 3 days of incubation.

**Conclusions:**

The current study provides a comprehensive characterization of the optimal conditions, which are essential to enhance lipase production in five *A. niger* isolates.

## Introduction

Lipases are enzymes that catalyze the reversible hydrolysis of triglycerides into fatty acid and glycerol. Some lipases catalyze both transesterification and enantioselective hydrolysis reactions (Zhang et al., 2001). The interest in lipase production has increased over the past several years due to their excellent catalytic properties (Sharmaa, Chistib & Banerjeea, 2001) and diverse industrial applications including detergents, esterification, pharmaceuticals, and production of biodiesel (Hasan, Shah & Hameed, 2006). Lipases occur widely in bacteria, yeasts, and fungi (Jaeger, Dijkstra & Reetz, 1999; Gaoa, Cao & Zhang, 2000; Dalmau et al., 2000). Many fungal genera have been broadly recognized as optimal lipase sources, *Aspergillus niger* is among the well-known lipase producers (Falony et al., 2006). It is necessary to study the characteristics of lipases because lipases from different sources exhibit different properties and they exhibit a wide range of numerous industrial applications.

Several studies have focused on identifying the optimal conditions for extracellular lipase production by fungi. The use of fungi as industrial producers has several advantages, including their ability to be used in a wide range of agricultural products, as a nutrient source, and for extracellular lipase production in fermentation cultures (Toscano et al., 2011). Numerous fungal species have been studied to investigate the mechanisms of lipase production. For example, soils polluted with waste oils or dairy/agricultural products have been shown to harbor many lipase-producing fungal species (Ko, Wang & Ann, 2005). Lipase produced by *Aspergillus* spp. has significant biotechnological applications. Therefore, several studies have been aimed at understanding the importance of fermentation factors, such as food, temperature, and fermentation time in lipase production. In addition, several *Aspergillus* lipases exhibit industrially important characteristics, such as pH and thermal stability; therefore, numerous highly specialized strategies have been developed for their purification (Sharmaa, Chistib & Banerjeea, 2001).

Several studies attempted to determine the nutritional requirements for optimal lipase production have showed that the concentration and the type of carbon and nitrogen sources, culture pH, and growth temperature affect lipase production (Elibol & Ozer, 2001). Fatty carbon sources are required to obtain a high yield of lipase. In addition, it has been shown that the most appropriate media for lipase production was not necessarily conducive for microbial growth (Adham & Ahmed, 2009). Furthermore, lipase production was increased by 1.8-fold under optimized conditions of culturing fungi in 1% sunflower oil, 0.8% glucose, and 0.8% peptone at 37 °C for 96 h with 200 rpm shaking. However, the requirement for a lipid carbon source remains essential for lipase production (Kaushik et al., 2006).

Furthermore, several studies have demonstrated that the extracellular production of lipase from different strains of *A. niger* is variable (Falony et al., 2006). The availability of DNA markers such as random amplified polymorphic DNA (RAPD) has enabled researchers to investigate genetic diversity among various species across natural populations. PCR-based RAPD markers have been widely used in assessing genetic variation within a species, including *Aspergillus* spp. (Midorikawa et al., 2008) In this technique, polymorphisms are detected in the entire genome, using established fingerprinting procedures. In this study, a robust and reproducible RAPD method was used to assist in determining whether genetically related strains correlate with their lipase production efficiency.

The objective of this study was to use molecular tools to identify fungal isolates that produce lipase by submerged fermentation (SmF) and determine their physiological and biochemical properties as well as the optimal conditions that facilitate lipase production with higher activity.

## Material and Methods

### Isolation of fungal strains

Oil seeds of pistachios, almonds, walnuts, cashews, sunflower, sesame, corn, and peanuts were collected from the local markets in different cities of Saudi Arabia (Dammam, Al-Khobar, Al-jubail, Hafr Al-Baten, and Al-Hassa). The seed surfaces were sterilized using 5% sodium hypochloride and dried. The seeds were incubated in Tween 80 agar as described by Malekabadia, Badoei-dalfarda & Karamia (2018) for 7 days at 27 °C. Then samples of grown fungi were purified on potato dextrose medium according to the method described by Ziaee, Zia & Goli (2018) and incubated for 5 days for further examination.

### Screening for lipase production by fungal isolates

For the screening of lipolytic fungi, the phenol red and tributyrin plate assays were applied to the isolates obtained from the Tween 80-treated samples. First, lipase activity was evaluated using phenol red agar medium to detect pH changes arising due to the hydrolysis of oil into fatty acids; typically, the pH of the medium reduces upon hydrolysis and is characterized by a yellow halo (Singh et al., 2006). The best isolates for enzymatic production were then selected to evaluate hydrolysis on tributyrin agar (TBA) medium to confirm the productivity of lipase that digests tributyrin, resulting in a colorless region as descried by Ayinla, Ademakinwa & Agboola (2017). Lipase production was determined by measuring the diameter of the yellow and clear zones (halos) that formed around the fungal growth in the phenol red and tributyrin agar media, respectively.

### DNA extraction and polymerase chain reaction (PCR)

Genomic DNA of the samples of *Aspergillus* isolates was extracted using the Wizard® Genomic DNA Purification Kit (Promega, WI, USA), according to the manufacturer’s protocol. Polymerase chain reaction (PCR) amplification reaction was performed in a final volume of 50 µl containing the following final concentrations of each component: 10 × PCR Top Taq Buffer, 25 mM MgCl_2_, 10 mM of each deoxynucleoside triphosphate, 2 U Top *taq* polymerase, and 10 µM each of 18SrRNA F: 5′-GCTTAATTTGACTCAACACGGGA-3′ and 18SrRNA R: 5′-AGCTATCAATCTGTCAATCCTGTC- 3′ primers to amplify 18SrRNA genes. The thermal cycling parameters were as follows: initial denaturation at 95 °C for 10 min, followed by 35 cycles of amplification at 95 °C for 1 min, annealing at 67.7 °C for 75 s, and extension at 72 °C for 2 min, with a final extension step of 5 min at 72 °C. In addition, lipase primers descried previously by Metwally et al. (2015) forward primer: 5′-ATGTTCTCTGGACGGTTTGGAGTG-3′; reverse primer: 5′-TTATAGCAGGCACTCGGAAATC-3′—were used to amplify the lipase gene of *A. niger* (Table 1). Identical thermal cycling parameters were used for the amplification of the lipase gene, except for the annealing temperature, which was set at 64.7 °C.

**Table 1:** Primers used to identify *Aspergillus spp.* and the presence of lipase gene.

**Gene target**	**Primer sequence (5′–3′)**	**Amplicon size (bp)**
18SrRNA F	5′-GCTTAATTTGACTCAACACGGGA-3′	1,655
18SrRNA R	5′-AGCTATCAATCTGTCAATCCTGTC-3′	
Lipase F	5′-ATGTTCTCTGGACGGTTTGGAGTG-3′	1,058
Lipase R	5′-TTATAGCAGGCACTCGGAAATC-3′	

### PCR purification and sequencing

The PCR product of the 18S rRNA gene was purified using QlAquick PCR purification kit (QIAGEN, Germany), following the manufacturer’s instructions. Purified amplicons were sequenced using the BigDye Terminator v3.1 Cycle Sequencing Kit (Applied Biosystems, Thermo Fisher Scientific, USA), with 1 µM primers (either 18SrRNA F or 18SrRNA R) and dideoxynucleotides (ddNTPs). The amplification products were purified using the BigDye X-Terminator purification kit (Applied Biosystems, Thermo Fisher Scientific, USA), according to the manufacturer’s protocol, and sequenced using Genetic Analyzer 3500 (Thermo Fisher Scientific, USA). Initial sequencing analysis was performed using 3500 Data Collection Software (Applied Biosystems, Thermo Fisher Scientific, USA), followed by further analysis using the basic local alignment search tool (BLAST), phymycodb, and MAFFT tools. The generated fasta sequence files were ultimately submitted to NCBI through Genbank.

### Random amplification polymorphic DNA (RAPD) analysis

The reactions were performed in 25 µl mixtures containing 25 ng extracted DNA, 1 µM primer, 20 mM Tris–HCl (pH 8.8), 2.5 mM MgCl_2_, 0.2 mM of each dNTP, and 1 U *Taq* DNA polymerase. Five primers were found suitable for RAPD analysis (Table 2) after they were tested in discriminate strain typing of *Aspergillus spp*. Amplifications were conducted with RAPD1, RAPD2, RAPD3, RAPD4, and RAPD5 primers under the following thermal cycling conditions: initial denaturation for 2 min at 94 °C, followed by 44 cycles of denaturation for 1 min at 94 °C, annealing for 2 min at 36 °C, and extension for 2 min at 72 °C, with a final extension step for 10 min at 72 °C. Amplified products were resolved in a 1.5% agarose gel and detected by ethidium bromide staining. DNA bands were analyzed using the UV-based detection method with the GelDoc XR imager (Bio-Rad Laboratories Inc., Hercules, CA). A 100-bp DNA ladder (Quick-Load® 100 bp DNA Ladder, QIAGEN, Germany) was used to estimate DNA fragment sizes.

**Table 2:** Primers used for random amplification polymorphic DNA (RAPD) analysis.

**Primer**	**Primer sequence (5′–3′)**	**Range of marker (kb)**
RAPD1	5′-CCACACTACC-3′	1.2–0.4
RAPD2	5′-CGGCCACTGT-3′	1.1–0.3
RAPD3	5′-CGGCCCCGGC-3′	1.5–0.3
RAPD4	5′-CGGAGAGCGA-3′	1.1–0.3
RAPD5	5′-GACGGAGCAG-3′	1.2–0.3

### Lipase enzyme production by submerged fermentation

Lipase production was examined using Tween 80 broth, as described previously (Ayinla, Ademakinwa & Agboola, 2017). The sterile medium was aseptically inoculated with 2 ml of 3 × 10^7^ spores/ml fungal conidial suspension that was prepared in a mineral salt solution. The culture was incubated at 25 ± 2°C for 5 days in a rotary shaker at 200 rpm. Post-incubation, the fungal broth was filtered using Whatman’s filter paper no. 1, and the top growth was dried in an oven at 60°C prior to weighing. The filtrate was centrifuged at 1,200 rpm for 30 min at 4°C, and the clear supernatant was subjected to the lipase assay using a spectrophotometer (SPECTRO UV–VIS DOUBL).

### Lipase enzyme activity assay

Lipase activity was assayed using the p-Nitrophenyl Palmitate (pNPP) hydrolysis method. The filtrate (100 µl) was incubated for 15 min at 30 °C with 800 µl of 0.25% polyvinyl alcohol (PVA) solution (pH: 6.5) and 100 µl of 3 mM pNPP solution in isopropanol. The reaction was terminated by adding 500 µl of 3 M HCl. Next, 500 µl of the centrifuged mixture was added to 1 ml of 2 M NaOH. Absorbance was measured in spectrophotometer (SPECTRO UV–VIS DOUBL) at 410 nm and the unit of the enzymatic activity (U) was determined from the release of 1 µM of pNPP per minute. A standard curve was used to measure enzyme activity, as described previously by Oliveira, Fernandes & Mariano (2014).

### Physiological and biochemical characterization

To determine the optimal parameters, cultures were incubated at different temperatures ranging from 15 to 45 °C (Sundar & Kumaresapillai, 2013), various pH levels ranging from 3 to 8.5 (Sethi, Tyagi & Anurag, 2016), various time periods of 3, 5, 7, 10, and 15 days (Niaz et al., 2014), different carbon sources; glucose, fructose, lactose, maltose, galactose, sucrose, and starch (Bindiya & Ramana, 2012), different nitrogen sources; peptone, yeast extract, beef extract, sodium nitrate, casein and potassium nitrate 6 (Ayinla, Ademakinwa & Agboola, 2017), various amounts—0.5, 1, 1.5, 2.5, and 3 ml—of spore suspension (3 × 10^7^ spores/ml) (Niaz et al., 2014), and different lipid sources—olive, sunflower, corn, soybean, palm, and castor oil—were used at various concentrations—1.5, 2.0, and 2.5 ml. Lipase activity was assayed in the filtered culture for all factors using submerged fermentation medium (Reshma & Shanmugam, 2013).

### Statistical analysis

Correlations between the enzyme activity and temperature, pH, incubation time, carbon source, nitrogen source, oil type, and inoculum volume were examined by conducting analysis of variance (ANOVA) using SPSS software version 23 (George & Mallery, 2016).

## Results

### Isolation of fungi from oil seeds

A total of 256 fungal isolates were obtained from culturing the following seeds: pistachio, cashew, almonds, corn, nut, and peanut, while sesame and sunflower seeds did not reveal any fungal growth. The highest number of fungal isolates among oil seeds was observed in pistachio, followed by cashew, almonds, corn, nut, and peanut seeds, in descending order. In addition, *Aspergillus* sp. were the most frequent isolates (*n* = 133, 52.00%), followed by *Penicillium* sp. (*n* = 63, 24.61%), *Alternaria* sp. (*n* = 32, 12.50%), and *Fusarium* sp. (*n* = 28, 10.94%, Table 3; Fig. S1).

**Table 3:** The frequency of fungi isolates collected from different oil seeds.

Isolates	*Aspergillus spp.*	*Penicillium spp.*	*Alternaria spp.*	*Fusarium spp.*	Total of isolates/seed type
Seed type					
Pistachio	80	28	21	–	129
Cashew	21	4	4	3	32
Almonds	21	–	6	–	27
Corn	–	1	–	25	26
Nut	–	26	–	–	26
Peanut	11	4	1	–	16
Total isolates/genus	133	63	32	28	256
Frequency of isolates	52.00%	24.61%	12.50%	10.94%	

### Screening of lipase production by fungal isolates

From each genus, one isolate was selected from each crop found in any of the five outlined cities (*n* = 35) to evaluate lipase production: *Aspergillus* (*n* = 13 isolates), *Penicillium* (*n* = 10 isolates), *Alternaria* (*n* = 7 isolates), and *Fusarium* (*n* = 5 isolates). Lipase production of the isolates was examined using the phenol red agar medium. *Aspergillus spp.* were found to be the highest lipase producers among various fungal genera, which produced less to no enzymes (Table S1). Therefore, *Aspergillus* isolates that were able to hydrolyze olive oil using the phenol red agar medium were selected for further analysis depending on their ability to hydrolyze tributyrin. The clear zones obtained using the tributyrin plate assay (Fig. S3) revealed that *A. niger* MH079049.1, *A. niger* MH111400.1, *A. niger* MH078565.1, *A. niger* MH078571.1, and *A. niger* MH111398.1 were the most efficient lipase producers (Table 4).

**Table 4:** Detection of the ability of fungal isolates to hydrolyze oil by measuring the yellow halo generated in the phenol red medium and the clear zone in the tributyrin agar medium.

***Aspergillus sp.***	**Seed type**	**Seed source**	**Halo of yellow (cm^2^)**	**Clear zone (cm^2^)**
*A. niger*MH078571.1	Almonds	Jubail	40.82	3.71
*A. niger*MH079049.1	Cashew	Dammam	48.36	3.52
*A. niger*MH111398.1	Pistachio	Dammam	33.91	3.45
*A. niger*MH078565.1	Bean	Dammam	42.08	3.39
*A. niger*MH111400.1	Pistachio	Hassa	45.84	3.27
*A. niger*MH078513.1	Pistachio	Hafr Al-Batin	28.89	2.64
*A. niger*MH111399.1	Pistachio	Khobar	28.26	2.26
*A. niger*MH057541.1	Pistachio	Jubail	37.68	1.82
*A. niger*MH111401.1	Bean	Khobar	40.19	1.7
*A. niger*MH078566.1	Almonds	Khobar	34.54	1.63
*A. niger*MH079063.1	Cashew	Jubail	40.19	1.44

### Lipase enzyme assay

Lipase activity was estimated using the spectrophotometric pNPP hydrolysis assay. *Aspergillus* isolates *A. niger* MH079049.1 (406.92 U/ml), *A. niger* MH078571.1 (390 U/ml), *A. niger* MH111400.1 (267.69 U/ml), *A. niger* MH078565.1 (255.38 U/ml), *and A. niger* MH111398.1 (196.92 U/ml) showed higher lipase activity than other isolates, such as *A. niger* MH111401.1 (35.38 U/ml), which showed minimal activity (Table 5). In addition, the results indicated no correlation between lipase activity and the growth of *Aspergillus* isolates.

**Table 5:** Lipase activity and dry weight of *Aspergillus* isolates.

***Aspergillus*Isolates**	**Lipase activity (U/ml)**	**Standard deviation**	**Dry weight**	**Standard deviation**
*A. niger*MH079049.1	406.92	±0.44	0.304	±0.010
*A. niger*MH078571.1	390	±2.70	0.696	±0.047
*A. niger*MH111400.1	267.69	±2.31	0.533	±0.030
*A. niger*MH078565.1	255.38	±0.44	0.22	±0.022
*A. niger*MH111398.1	196.92	±2.91	0.329	±0.012
*A. niger*MH079063.1	150.77	±1.54	0.398	±0.021
*A. niger*MH111399.1	135.38	±1.18	0.137	±0.015
*A. niger*MH078513.1	121.54	±1.60	0.277	±0.012
*A. niger*MH078566.1	110	±0.89	0.368	±0.037
*A. niger*MH057541.1	86.92	±1.54	0.128	±0.042
*A. niger*MH111401.1	35.38	±2.35	0.141	±0.009

### Molecular characterization of *Aspergillus spp.* isolates

Isolated *Aspergillus spp.* were identified using 18srRNA gene sequencing. All 11 *Aspergillus* strains that were tested in the current study were identified as *A. niger*. The sequences were submitted to GenBank under the accession numbers MH111398.1, MH111399.1, MH057541.1, MH111400.1, MH078513.1, MH078565.1, MH111401.1, MH078566.1, MH078571.1, MH079049.1, and MH079063.1. In addition, the presence of the lipase gene was verified in all 11 strains by PCR amplification (Fig. S5).

**Figure 1:**
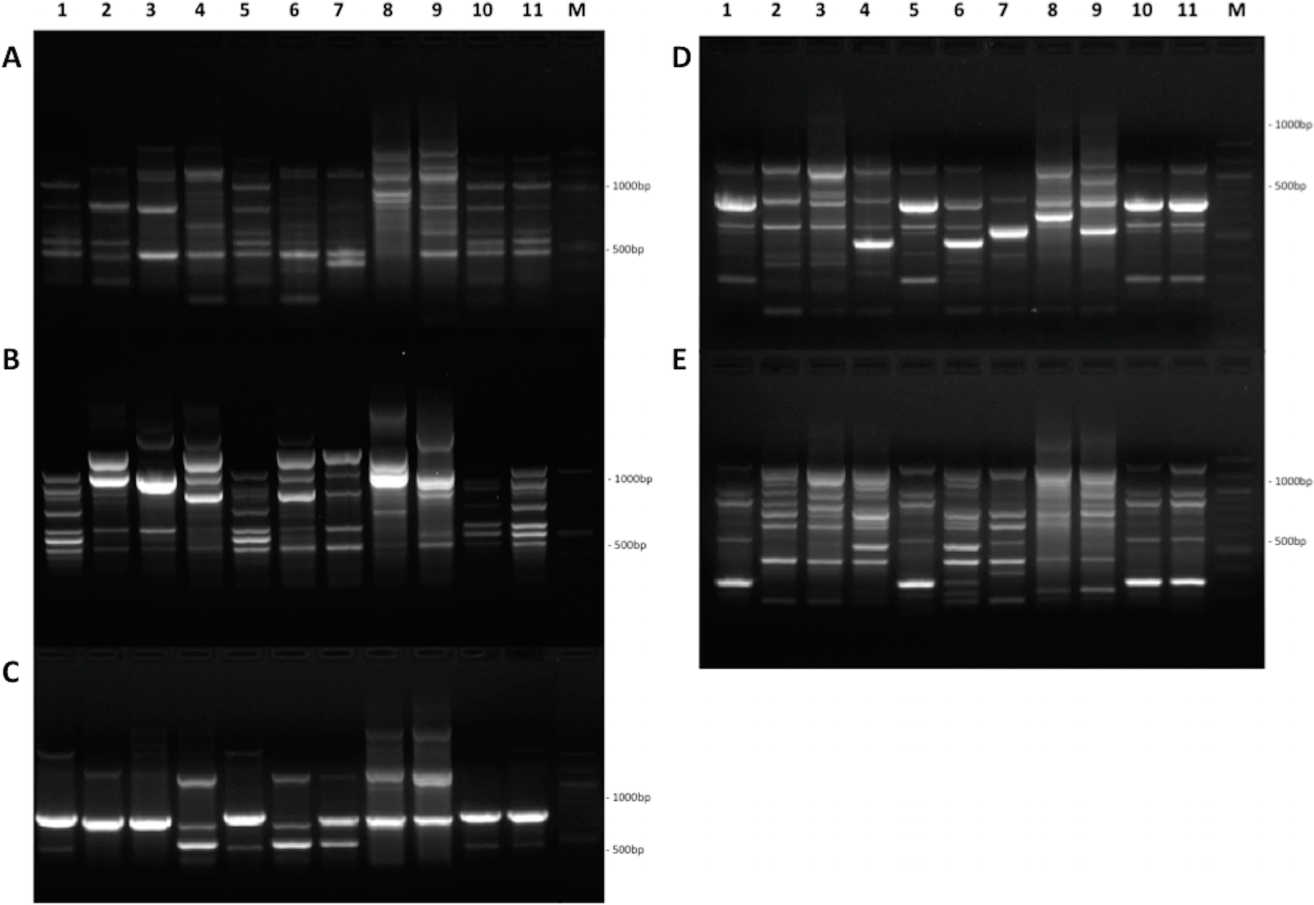
Comparison of RAPD patterns among Aspergillus niger strains. DNA fragments amplified from DNA samples of 11 Aspergillus niger strains using each of the five different RAPD primers: (A) RAPD1, (B) RAPD2, (C) RAPD3, (D) RAPD4, and (E) RAPD5. Samples were oriented in the following order: MH111398.1, MH111399.1, MH057541.1, MH111400.1, MH078513.1, MH078565.1, MH111401.1, MH078566.1, MH078571.1, MH079049.1, and MH079063.1. Numbers on the right (M) indicate sizes (bp) of the components of a 1-kb ladder (Quick-Load 100 bp DNA Ladder, QIAGEN, Germany).

**Figure 2:**
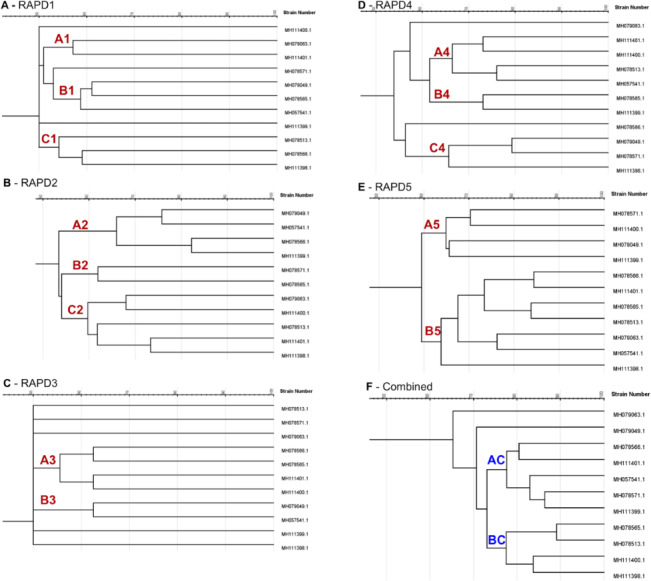
Dendrogram illustration of the genetic fingerprint and relationships between A. niger isolates developed using unweighted pair group method with arithmetic average analysis from RAPD data. From A to E, A1–B5 and C1–C4 indicate clusters at the level of 60% and 75% similarity in individual RAPD analysis, respectively. In F, AC and BC indicate clusters at 75% genetic similarity in the combined analysis of the random polymorphic amplicons.

RAPD analysis using five primers to amplify random regions of 11 *A. niger* strains genomic DNA yielded 285 fragments that could be analyzed. All of the five primers amplified fragments across the 11 studied strains, with the number of amplified fragments varying from two (Fig. 1C) to eight (Fig. 1E) and ranging from 200 bp to 2,500 bp. Of the 285 amplified bands, five were polymorphic with an average of one polymorphic fragment per primer. Polymorphism ranged from 39.77% for (Fig. 1A) to a maximum of 53.63% (Fig. 1B), with an average of 45.99%. Only 1 (Fig. 1B) out of 5 primers showed more than 50% polymorphism. The extent of polymorphism detected among the isolates, as revealed by RAPD, is represented in (Figs. 1A to 1E). A dendrogram based on UPGMA analysis grouped the 11 *A. niger* strains into main clusters. A1 to B5 and C1 to C4 indicate clusters at the level of 60% and 75% similarity in individual RAPD analysis, respectively (Figs. 2A to 2E); whereas, AC and BC indicate clusters with 75% genetic similarity in the combined analysis of the random polymorphic amplicons (Fig. 2F).

### Physiological and biochemical characterization of lipase production in *Aspergillus* isolates

Five *Aspergillus* isolates were selected as the highest lipase producers based on the results of the lipase production test using the tributyrin and lipase activity tests. The isolates *A. niger* MH111398.1, *A. niger* MH111400.1, *A. niger* MH078565.1, *A. niger* MH078571.1, and *A. niger* MH079049.1 were examined in a submerged fermentation culture (Smf) to measure their enzymatic activity.

### Effect of temperature

**Figure 3:**
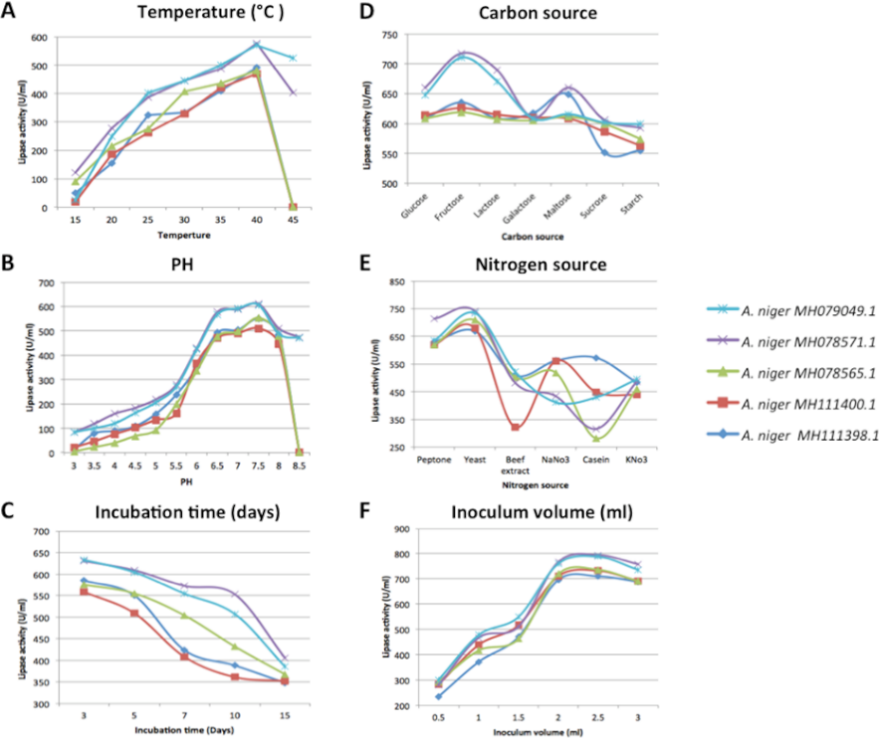
Measurements of lipase enzymatic activity (U/ml) on the submerged fermentation culture (Smf) including the following factors: (A) temperature, (B) pH, (C) incubation time, (D) carbon source, (E) nitrogen source, and (F) inoculation volume.

The metabolic activity at 0 °C is low, which results in the inhibition of lipase activity. At 40 °C, the isolate that exhibited the highest lipase activity was *A. niger* MH078571.1 (575.9 U/ml), while the *A. niger* MH111400.1 exhibited the lowest lipase activity (470.51 U/ml). At 45 °C, two out of five isolates were able to grow in the submerged culture–*A. niger* MH078571.1 (403.3 U/ml) and *A. niger* MH079049.1 (524.87 U/ml)–whereas, the other isolates were not able to grow under these conditions. However, lipase production at this temperature was lower than that at 40 °C. Therefore, the optimum temperature was fixed at 40 °C (Table S2; Fig. 3A). The measurement of the dry weight of all fungal isolates showed that a temperature of 15 °C was the optimal for fungal growth in the submerged fermentation culture, but it was a poor catalyst for lipase production. In addition, mycelium growth was measured to determine the extent of fungal growth. The mycelium diameter increased as the temperature increased up to 30 °C, and then gradually began to decrease as the temperature further increased.

### Effect of pH

The results of the present investigation showed that increasing the acidity of the culture plays a role in inhibiting lipase production and increasing alkalinity; a pH of 7.5 yielded optimal lipase production for the five tested isolates, with the highest lipase activity exhibited by *A. niger* MH078571.1 (610.77 U/ml) and *A. niger* MH079049.1 (606.15 U/ml). Most isolates had lost their ability to grow at pH 8.5, except two isolates—*A. niger* MH078571.1 and *A. niger* MH079049.1—both of which were able to grow and produce lipase under alkaline conditions of the culture; however, their lipase yields were low. Therefore, a pH of 7.5 was selected as the optimum pH for lipase production for the selected isolates (Table S3; Fig. 3B).

### Effect of incubation time

The results in Table S4 and Fig. 3C demonstrate that the optimal duration for the highest lipase production was 3 days. The longer the incubation of the isolates, the lesser the lipase yield. The lipase activity of the isolates reached minimum values after 15 days of incubation. For instance, *A. niger* MH111398.1 showed low lipase activity (348.21 U/ml) after 15 days. In contrast, mycelium growth density in the submerged culture as well as on surface of the petri dishes increased with an increase in the incubation duration; the isolate yielding the highest density after an incubation period of 15 days was the dry mycelium of *A. niger* MH079049.1 (1.246 g). Therefore, the duration of incubation was fixed to study the physiological factors after 3 days of incubation.

### Effect of carbon source

Five fungal isolates were grown on media containing olive oil as the sole source of energy without the presence of a carbon source to stimulate lipase production. However, the presence of a carbon source contributes to improvement of fermentation and subsequently increases cellular metabolism. Thus, increasing cellular metabolism contributes to increased fungal lipase activity. Different carbohydrates acted as monovalent sources for optimal enzymatic production; fungi produce low levels of lipase in the presence of starch and sucrose. Fructose exhibited the highest positive impact on lipase production in *A. niger* MH078571.1 (717.44 U/ml), followed by *A. niger* MH079049.1 (710.26 U/ml) (Table S5; Fig. 3D). These results showed a correlation between mycelium growth and lipase production in the presence of fructose.

### Effect of nitrogen source

Yeast extract was shown to be the most effective nitrogen source, and the highest value of 741.54 U/ml was obtained for *A. niger* MH078571.1, followed by *A. niger* MH079049.1 (734.36 U/ml). The results outlined in Table S6 and Fig. 3E show that mycelium growth and linear fungal growth were the highest in the presence of peptone; the highest linear growth value was 7.6 cm for *A. niger* MH078571.1 and the highest mycelium growth value was 1.309 g for *A. niger* MH079049.1. Casein and potassium nitrate exhibited the least effect on mycelium growth and linear growth.

### Effect of inoculum volume

Various amounts—0.5, 1, 1.5, 2.5, and 3 ml—of spore suspension (3 × 10^7^ spores/ml) inoculated on the submerged fermentation culture revealed that the lowest values of lipase production were with an inoculum volume of 0.5 ml for all isolates. On the other hand, lipase production increased as the inoculum volume increased, until reaching the highest value at 2.5 ml; (794.62 U/ml) obtained for *A. niger* MH078571.1. Inoculum volumes higher than 2.5 ml decreased both lipase production and mycelium growth (Table S7; Fig. 3F).

### Effect of different lipid sources

The examination of different oil sources, such as olive, sunflower, corn, soybean, palm, and castor oils at volumes of 1.5, 2.0, and 2.5 ml on lipase activities using the submerged fermentation medium revealed a 2.0 ml/L of palm oil as the optimal oil type and concentration for lipase production (Table S8). The optimal enzymatic production for all of the oil types (olive, sunflower, castor, and palm oils) was at 2% and the increase in the concentration up to 2.5% showed decreased lipase production in all of the tested strains, except for corn oil, which showed optimal production at 2.5%.

## Discussion

This study examined a total of 35 fungal isolates derived from oilseeds that were collected from cities located in the Eastern province of Saudi Arabia. These isolates include *Aspergillus spp.*, *Alternaria spp.*, *Penicillium spp.,* and *Fusarium spp. Aspergillus* strains exhibited the highest lipase production; 11 *Aspergillus* strains were selected for this study, of which, five of the highest lipase producers were selected to examine enzymatic activity and identify the optimal growth conditions, such as temperature, pH, incubation time, carbon source, nitrogen source, lipid source, and inoculum amount. The examination of these isolates in a submerged fermentation culture (Smf) revealed the following optimal conditions: 40 °C, pH 7.5, 1% fructose as the carbon source, 1% yeast extract as the nitrogen source, 2% palm oil, 2.5 × 10^7^ spores/ml spore suspension, and an incubation period of 3 days.

Many fungal species can produce lipase, and the most important commercial lipase-producing fungi belong to the genera *Rhizopus, Aspergillus, Penicillium, Geotrichum, Mucor,* and *Rhizomucor*. Fungal lipase is thermally more stable than bacterial lipase, and it contains several properties that are preferable for contemporary applications, such as the fermentation of closed systems or batch cultures and the low-cost enzyme extraction. Lipase converts lipids in media into fatty acids and glycerol, causing the pH to decrease. Therefore, the lipase test was conducted by using a phenolic medium with phenol red as an indicator. The diameter of the colored region increases proportionally with changes in the amount of lipids that are metabolized (Narasimhan & Bhimba, 2015). In addition, tributyrin is a fatty substance that can be digested by lipases, thus rendering it the preferred assay for testing lipase-producing microorganisms. Therefore, lipase production was investigated using tributyrin agar to observe the distinct halo caused by the digestion of the substance by the lipases (Narasimhan & Bhimba, 2015).

*A. niger* is one of the most effective lipase producers (Falony et al., 2006). Moreover, studies have shown that the extracellular production of lipase in *A. niger* differs across various strains. Molecular studies are essential in understanding the factors that contribute to variations in enzymatic production among *A. niger* strains. In this study, the lipase gene was amplified in each of the 11 *A. niger* strains that produced lipases. Further experiments are required to estimate the expression of the lipase gene among the 11 isolates. In addition, sequencing of the lipase gene in all strains is necessary to determine the presence of variants in the gene that might contribute to differences in lipase production.

To gain insight into this genetic variability, we conducted RAPD-based marker analyses. Analyzing RAPD banding patterns in variety of *A. niger* isolates is expected to provide valuable knowledge regarding the genetic relationships that are present within lipase-producing strains. This may aid in determining whether strains from any one genetic subgroup are more frequently correlated with the high production of lipase enzymes. Further analysis that utilizes dendrograms based on RAPD fingerprinting with RAPD primers was conducted in an effort to indicate the isolates that are more closely related to each other and determine their degree of genetic relatedness. Dendrograms based on RAPD fingerprinting revealed three of the top five lipase enzyme-producing strains—*A. niger* MH078565.1, *A. niger* MH111400.1, and *A. niger* MH111398.1 with 75% genetic similarity. However, the top two strains—*A. niger* MH079049.1 and *A. niger* MH078571.1—showed low genetic similarity. The RAPD banding patterns using these five primers compared the genomic DNA of the isolates as a whole and not necessarily regions of their genomes that correlate with lipase enzyme production.

Lipase production by fungi varies according to the strain, growth medium, culture conditions, pH, temperature, and carbon and nitrogen sources (Treichel, Oliveira & Mazutti, 2010; Liu et al., 2015; Amoah et al., 2016). SmF is the most widely used method to produce industrial enzymes by more than 75%. This method has several advantages over solid-state fermentation culture (SSF), such as easy and large-scale procurement of the lipase and controlling the factors that affect the production (Sundar & Kumaresapillai, 2013; Oliveira, Fernandes & Mariano, 2014). Eleven *Aspergillus* isolates were tested for their ability of producing lipase using SmF as a growth medium to obtain lipase in culture.

Temperature significantly affects fungal growth and metabolic activity. Isolates showed high heat tolerance at different temperatures with the highest enzymatic production occurred at 40 °C. At 45 °C, isolates were unable to grow or produce the enzyme due to the mutilation of cellular proteins (Singh & Mukhopadhyay, 2012), except for *A. niger* MH078571 and *A. niger* MH079049, which exhibited the potential of lipase production and were resistant to high temperatures (Oliveira, Watanabe & Vargas, 2012; Niaz et al., 2014). Higher temperatures that enhance lipase synthesis and reduce *A. niger* growth have been reported previously (Nema et al., 2019). In addition, different temperatures have been reported for the maximum lipase activity such as at 30 °C for *P. chrysogenum*, which is the natural growth temperature of fungi (Ramani et al., 2010), at 37 °C for *A. terreus* (Gulati, Saxena & Gupta, 1999) and *R. oryzae* (Yuzbashev, Yuzbasheva & Vibornaya, 2012). Studies have revealed a correlation between enzymes yield with high temperatures, which may be attributed to the increase in the rate of enzymatic reactions and therefore here increase the lipase production (Jurjen Frankena et al., 1986).

Lipase activity at the various acidic and alkaline levels was found, but the optimal lipase activity was at pH 7.5, due to physiological and metabolic adaptation of fungi that contribute to their survival and lipase production. These adaptive properties include maintaining stability based on ionic membrane permeability, the storage capacity of cytoplasm, ion transport (Na^+^, K^+^, and H^+^), and modification of fatty membranes (Salihua & Alama, 2015). Some microorganisms have corresponded with this result for lipase production; for instance, *R. oryza* (Yuzbashev, Yuzbasheva & Vibornaya, 2012) and *A. terreus* (Mahmoud et al., 2015). Also, at pH 7.0, lipase production was maximum in *A. nidulans* (Niaz et al., 2014), *Cryptococcus sp.* (Maia et al., 2001), *P. chrysogenum* (Kumar et al., 2011), and *Burkholderia multivorans* (Gupta, Sahai & Gupta, 2007). Although the culture pH of 7.0 is neutral and optimal for fungal growth, various pHs significantly affect the enzymatic processes by controlling nutrient transport across the cell membrane (Moon & Parulekar, 1991).

The duration of the incubation period also plays a pivotal role in lipase synthesis. In consistent with previous studies, 3 days (72 h) constituted the optimal incubation period at 40 °C and pH 7.5 (Kumar et al., 2011; Niaz et al., 2014; Mukhtar, Hanif & Rehman, 2015). However, a study found that maximum enzymatic production occurred after an incubation period of 96 h, while the incubation period required by *R. arrhizus* and *A. niger* to reach maximum enzymatic production was 24 h (Yang et al., 2005). Increasing the incubation period beyond 3 days led to decreased enzyme activity, and fungi were less productive after 15 days of incubation. This decrease may be due to nutrient depletion and metabolic byproducts (inhibitors) (Sharma et al., 2016).

Carbohydrate sources were considered the main factors influencing fungal growth and their metabolic activity. The preference of the selected fungi was tested for monoclonal, bilateral, or multiple sugars. The maximum production of lipase occurred in the presence of mono-fructose sugar, which can be easily absorbed by the fungus. Glucose is also a monoclonal sugar that can enhance the fungi growth; however, it has been shown to reduce lipase activity (Burkert, Maugeri & Rodrigues, 2004). In descending order, the most effective sources were lactose, glucose, maltose, galactose, sucrose, and starch. This result is consistent with those of Iftikhar, Niaz & Jabeen (2012). While glucose was found to be the optimal carbon source (Adham & Ahmed, 2009; Niaz et al., 2014), Bacha et al. (2018) found that maximum lipase production occurred in the presence of xylose and lactose followed by galactose, starch, glucose, and sucrose. However, other study conducted on *A. niger* found that the optimal lipase production was obtained using 80 Tween as a source of carbon (Sundar & Kumaresapillai, 2013). Carbon source influences different enzyme production considerably and different microorganisms have been reported to utilize different carbon sources for their growth and metabolism.

Various nitrogen sources affecting enzymatic production were studied to examine the role of amino acids in cellular metabolic activity. Our results showed that the optimal source was yeast extract followed by peptone, meat extract, calcium nitrate, and sodium nitrate, in descending order of efficiency, which concurs with the results obtained by Bacha et al. (2018), who established that the best nitrogen source was yeast extract, followed by tryptone and ammonium nitrate. Peptone was found to be an optimal nitrogen source in the study conducted by Mukhtar, Hanif & Rehman (2015) and Sharma et al. (2016), while sodium nitrate was the least stimulating, which is consistence with our results. This is could be explained by the inhibitory effect when using inorganic nitrogen sources on both growth and lipase activity (Maldonado et al., 2016). Furthermore, ammonium sulphate was the most productive catalyst for lipase production in *A. niger* (Sundar & Kumaresapillai, 2013). Upon testing ammonium molybdates, ammonium nitrate, and urea as nitrogen sources, all tested fungal isolates could not grow either in submerged or solid media because the concentrations tested from these sources were likely to be cytotoxic, thus leading to the deformation of cellular proteins and mitigating fungal growth (Sharma et al., 2016).

The appropriate inoculum volume is an important key factor to balance the amount of nutrient substances present in the submerged environment for optimal fungal growth and lipase production. A linear increase in lipase production and inoculum volume was observed until the maximum production was reached at 2.5 × 10^7^ ml/spores. Our results were consistent with those of Iftikhar, Niaz & Jabeen (2012), while Niaz et al. (2014) found that the optimum volume is 2 ml. However, our results demonstrated that lipase production decreases when the inoculums volume is increased to 3 × 10^7^ ml/spores. This decrease in lipase production could be due to the large mass of mycelium that uses large amounts of substrates (fatty substances) as nutrients, leaving the non-fatty part of the food material to support their survival (Niaz et al., 2014).

Fatty sources play a significant role as a catalyst of fungal activity, thereby enhancing lipase production. Different concentrations (1.5%, 2%, and 2.5%) were studied for different oils, and the optimal concentration was for palm oil to stimulate lipase production with 2%. This is due to the hydrolysis of medium-chain fatty acids (C12) found in palm oil that can be metabolized more rapidly than long-chain saturated fatty acids, such as olive oil (>C20) (Cassiday, 2017).

## Conclusions

In this study, 35 fungal isolates were obtained from different types of oil seeds, and 11 isolates belonging to the genus *Aspergillus* were analyzed for lipase production. All the tested isolates were identified as *A. niger* using 18s rRNA sequencing. Genetic diversity was evaluated among the 11 *A. niger* isolates using random amplified polymorphic DNA (RAPD) primers. The tributyrin test and the lipase activity assay identified five strains of *A. niger* as high lipase producers, and their optimal enzyme activities were studied. The optimal conditions for lipase production were as follows: 40 °C, pH 7.5, 1% fructose as the carbon source, 1% yeast extract as the nitrogen source, 2% palm oil, 2.5 × 107 spores/ml suspension, and 3 days of incubation. A comprehensive characterization of *A. niger* isolates and lipase production conditions for these isolates will be essential to enhance lipase production.

##  Supplemental Information

10.7717/peerj.9425/supp-1 Figure S1 Images of fungal isolates Images of fungal isolates for each genus isolated from different kinds of oil seeds, (A) Under microscope (40X) and (B) on a petri dish.

10.7717/peerj.9425/supp-2 Figure S2 The difference between lipase The difference between lipase producing and non-producing isolates on phenol red agar medium, (A) yellow zone indicates lipase production and (B) the absent of the yellow zone indicting no lipase production.

10.7717/peerj.9425/supp-3 Figure S3 The colorless halo The colorless halo is formed by the hydrolysis of Tributyrin using commercial lipase enzyme (A) and by *Aspergillus* lipase production (B).

10.7717/peerj.9425/supp-4 Figure S4 (A) Under microscope (40X) and (B) on a petri dish photo of the 11 *Aspergillus sp.* Isolates producing lipase enzyme and examined in the current study

10.7717/peerj.9425/supp-5 Figure S5 Lipase gene amplification of DNA extracted from the 11 *Aspergillus niger* strains Amplicons were separated on agarose gels stained with ethidium bromide. Lane 1 through lane 11 are *A. niger* strains; MH111398.1, MH111399.1, MH057541.1, MH111400.1, MH078513.1, MH078565.1, MH111401.1, MH078566.1, MH078571.1, MH079049.1, MH079063.1 represent respectively. Lane 12 is 1-kb ladder (Quick-Load® 100 bp DNA Ladder, QIAGEN, Germany).

10.7717/peerj.9425/supp-6 Table S1 Detection of (*n* = 35) fungal isolates ability to hydrolyze olive oil using the phenol red medium

10.7717/peerj.9425/supp-7 Table S2 The effect of range of temperatures on the enzymatic activity of the 5 highest lipase producers of *Aspergillus sp.* Isolates

10.7717/peerj.9425/supp-8 Table S3 The effect of range of pH on the enzymatic activity of the 5 highest lipase producers of *Aspergillus sp.* Isolates

10.7717/peerj.9425/supp-9 Table S4 The effect of various incubation times on the enzymatic activity of the 5 highest lipase producers of Aspergillus sp. Isolates

10.7717/peerj.9425/supp-10 Table S5 The effect of different carbon sources on the enzymatic activity of the 5 highest lipase producers of *Aspergillus sp.* Isolates

10.7717/peerj.9425/supp-11 Table S6 The effect of different nitrogen sources on the enzymatic activity of the 5 highest lipase producers of *Aspergillus sp.* Isolates

10.7717/peerj.9425/supp-12 Table S7 The effect of various inoculum volume on the enzymatic activity of the 5 highest lipase producers of *Aspergillus sp.* Isolates

10.7717/peerj.9425/supp-13 Table S8 The effect of various oil types on the enzymatic activity of the 5 highest lipase producers of *Aspergillus sp.* Isolates

10.7717/peerj.9425/supp-14 Supplemental Information 14 The effect of range of temperatures on the enzymatic activity of the 5 highest lipase producers of *Aspergillus sp.* Isolates

10.7717/peerj.9425/supp-15 Supplemental Information 15 The effect of range of pH on the enzymatic activity of the 5 highest lipase producers of *Aspergillus sp.* Isolates

10.7717/peerj.9425/supp-16 Supplemental Information 16 The effect of various incubation times on the enzymatic activity of the 5 highest lipase producers of *Aspergillus sp.* Isolates

10.7717/peerj.9425/supp-17 Supplemental Information 17 The effect of different carbon sources on the enzymatic activity of the 5 highest lipase producers of *Aspergillus sp.* Isolates

10.7717/peerj.9425/supp-18 Supplemental Information 18 The effect of different nitrogen sources on the enzymatic activity of the 5 highest lipase producers of *Aspergillus sp.* Isolates

10.7717/peerj.9425/supp-19 Supplemental Information 19 The effect of various inoculum volume on the enzymatic activity of the 5 highest lipase producers of *Aspergillus sp.* Isolates

10.7717/peerj.9425/supp-20 Supplemental Information 20 The effect of various oil types on the enzymatic activity of the 5 highest lipase producers of *Aspergillus sp.* Isolates

## Additional Information and Declarations

### DNA Deposition

The following information was supplied regarding the deposition of DNA sequences: The sequences are available at GenBank: MH111398.1, MH111399.1, MH057541.1, MH111400.1, MH078513.1, MH078565.1, MH111401.1, MH078566.1, MH078571.1, MH079049.1, and MH079063.1. In addition, the presence of the lipase gene was verified in all 11 strains by PCR amplification is shown in Fig. S5.

### Data Availability

The following information was supplied regarding data availability: The raw measurements are available in the Supplemental Files.
