# Supplementary material for: Molecular, physiological, and biochemical characterization of extracellular lipase production by Aspergillus niger using submerged fermentation
Source: PeerJ. 2020 Jul 7;8:e9425. doi: 10.7717/peerj.9425 (PMC7350912; doi:10.7717/peerj.9425)
Supplement: Figure S1 — Images of fungal isolates for each genus isolated from different kinds of oil seeds, (A) Under microscope (40X) and (B) on a petri dish. [file peerj-08-9425-s001.pdf]

A	B
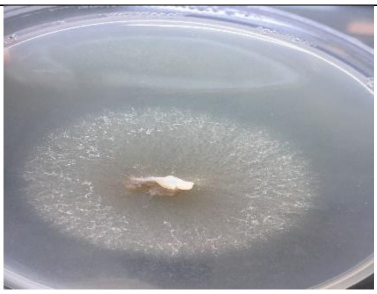 *Fusarium sp.*	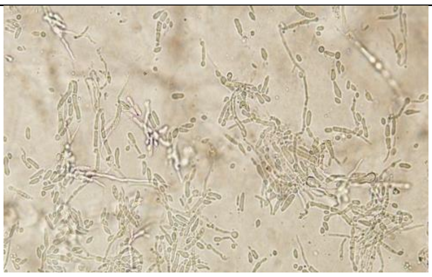 *Fusarium sp.*
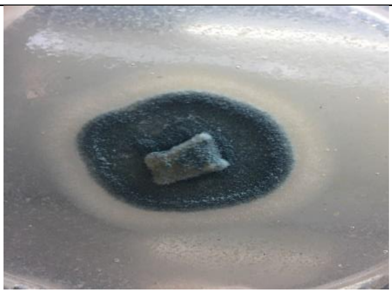 *Penicillium sp.1*	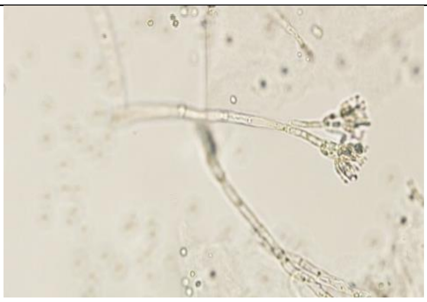 *Penicillium sp.1*
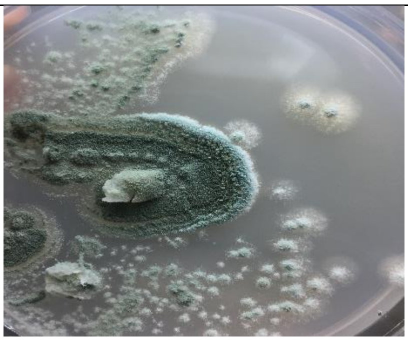 *Penicillium sp.2*	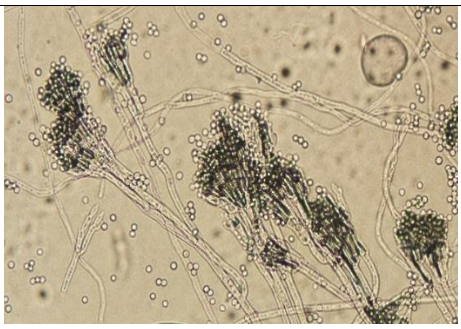 *Penicillium sp.2*
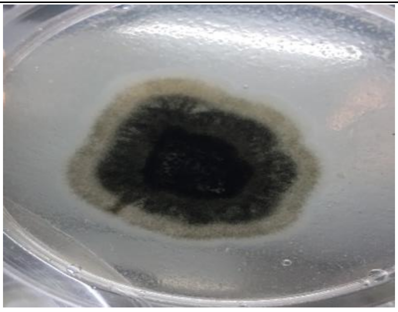 *Alternaria sp.*	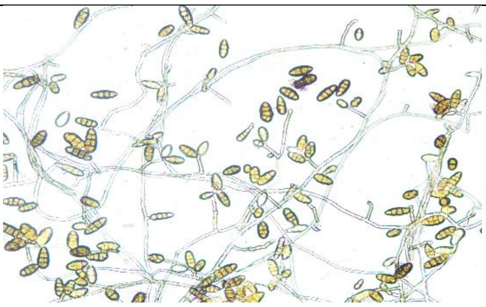 *Alternaria sp.*

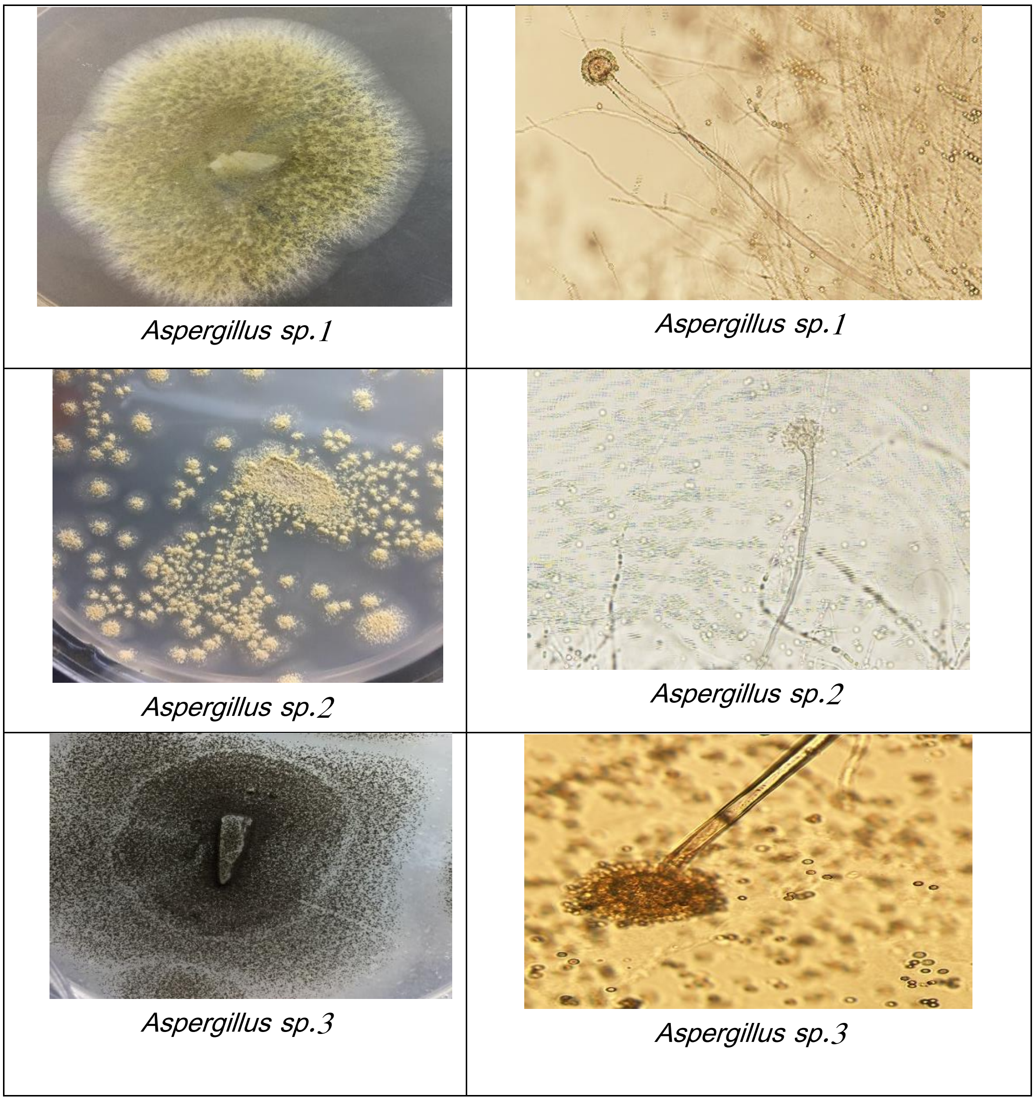

Figure 1. Images of fungal isolates for each genus isolated from different kinds of oil seeds, (A) Under microscope (40X) and (B) on a petri dish.
