# Supplementary material for: Molecular, physiological, and biochemical characterization of extracellular lipase production by Aspergillus niger using submerged fermentation
Source: PeerJ. 2020 Jul 7;8:e9425. doi: 10.7717/peerj.9425 (PMC7350912; doi:10.7717/peerj.9425)
Supplement: Figure S2 — The difference between lipase producing and non-producing isolates on phenol red agar medium, (A) yellow zone indicates lipase production and (B) the absent of the yellow zone indicting no lipase production. [file peerj-08-9425-s002.pdf]

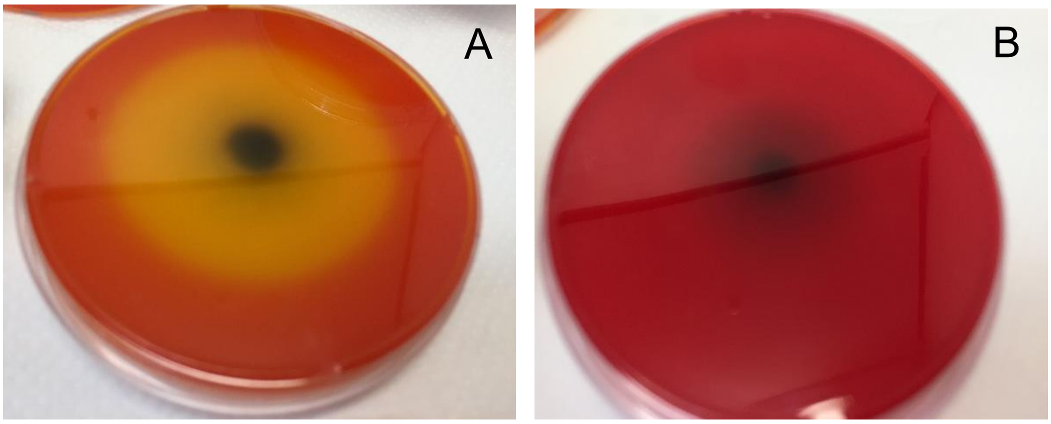

Figure 2. The difference between lipase producing and non- producing isolates on phenol red agar medium, (A) yellow zone indicates lipase production and (B) the absent of the yellow zone indicting no lipase production.
