# Supplementary material for: Molecular, physiological, and biochemical characterization of extracellular lipase production by Aspergillus niger using submerged fermentation
Source: PeerJ. 2020 Jul 7;8:e9425. doi: 10.7717/peerj.9425 (PMC7350912; doi:10.7717/peerj.9425)
Supplement: Figure S3 — The colorless halo is formed by the hydrolysis of Tributyrin using commercial lipase enzyme (A) and by Aspergillus lipase production (B). [file peerj-08-9425-s003.pdf]

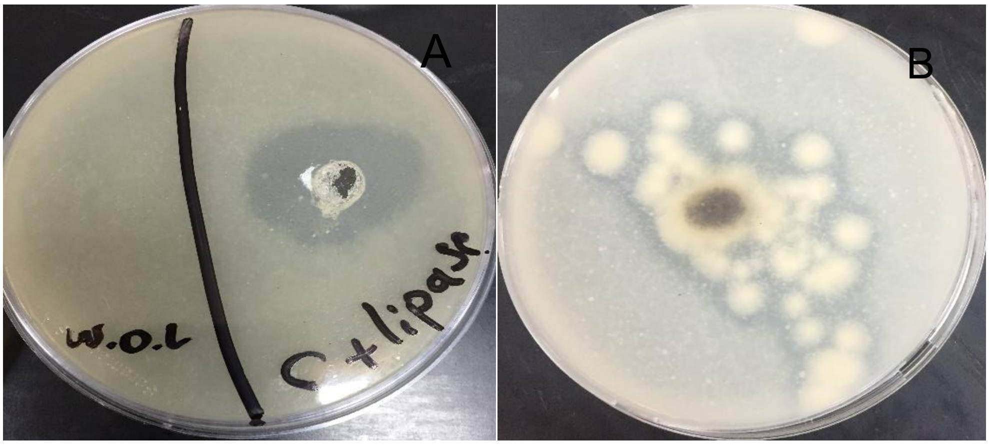

Figure 3. The colorless halo is formed by the hydrolysis of Tributyrin using commercial lipase enzyme (A) and by *Aspergillus* lipase production (B).
