# Supplementary material for: Molecular, physiological, and biochemical characterization of extracellular lipase production by Aspergillus niger using submerged fermentation
Source: PeerJ. 2020 Jul 7;8:e9425. doi: 10.7717/peerj.9425 (PMC7350912; doi:10.7717/peerj.9425)
Supplement: Figure S4 [file peerj-08-9425-s004.pdf]

(A) Microscope (40X)	(B) Petri dish
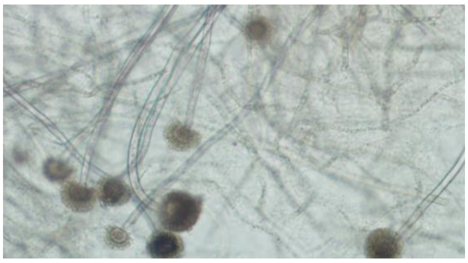 *A. niger* MH111398	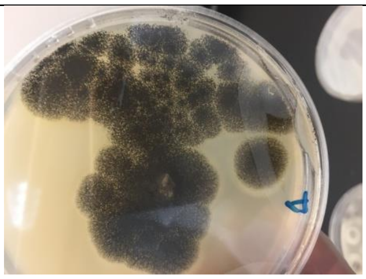 *A. niger* MH111398
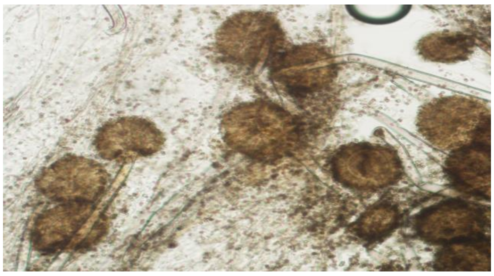 *A. niger* MH111399	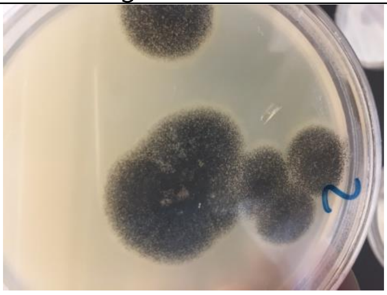 *A. niger* MH111399
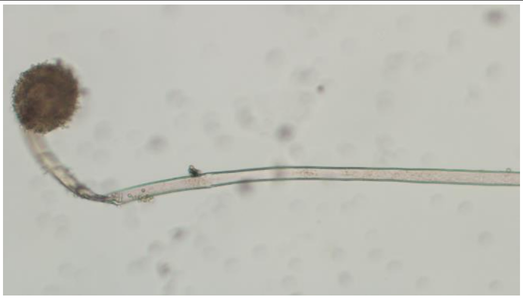 *A. niger* MH057541	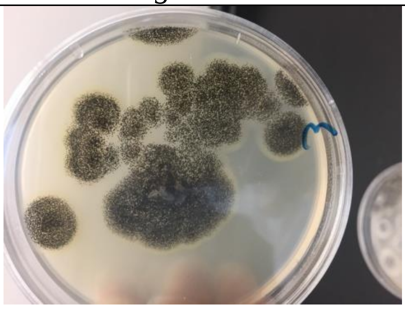 *A. niger* MH057541
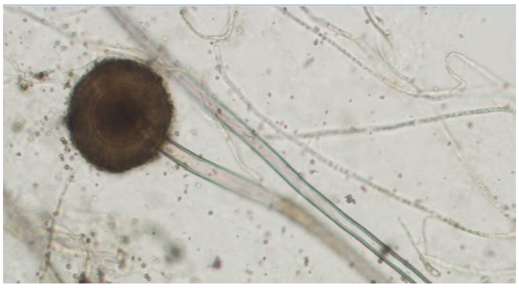 *A. niger* MH111400	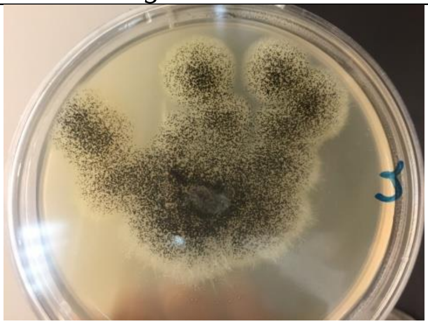 *A. niger* MH111400

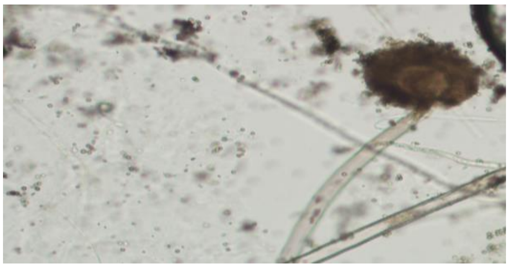<br>*A. niger MH078513*	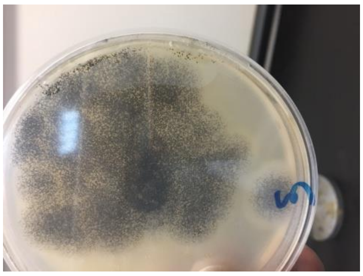<br>*A. niger MH078513*
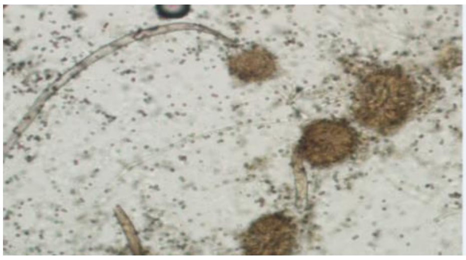<br>*A. niger MH078565.1*	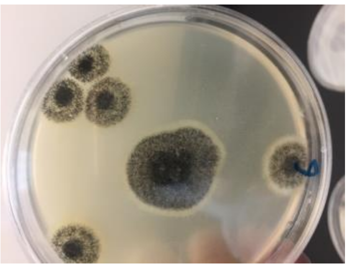<br>*A. niger MH078565.1*
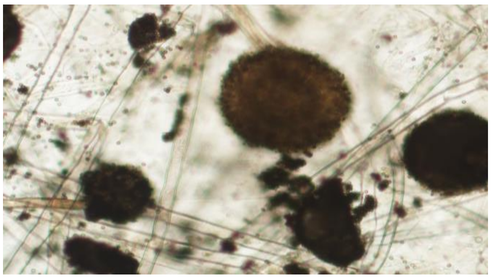<br>*A. niger MH111401.1*	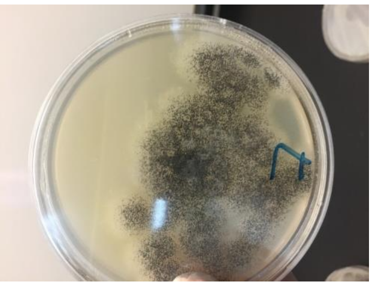<br>*A. niger MH111401.1*
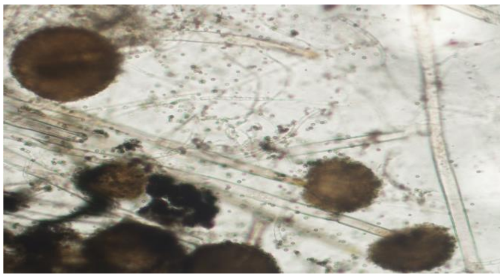<br>*A. niger MH078566.1*	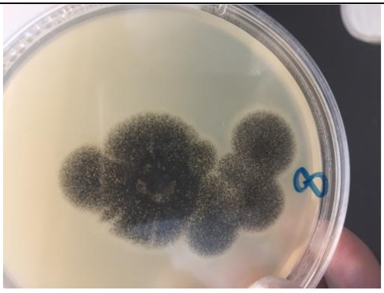<br>*A. niger MH078566.1*
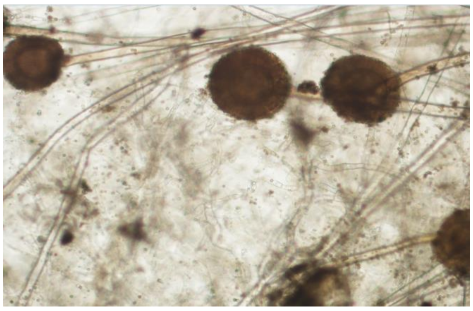<br>*A. niger MH078571.1*	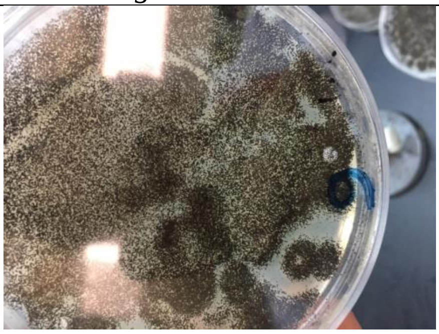<br>*A. niger MH078571.1*

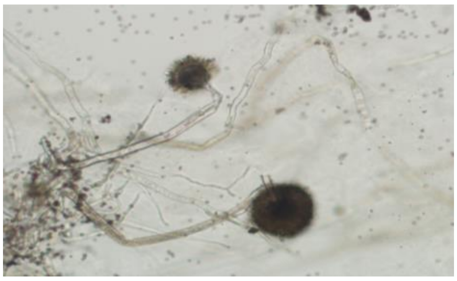<br>*A. niger MH079049.1*	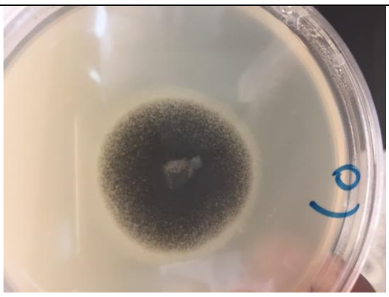<br>*A. niger MH079049.1*
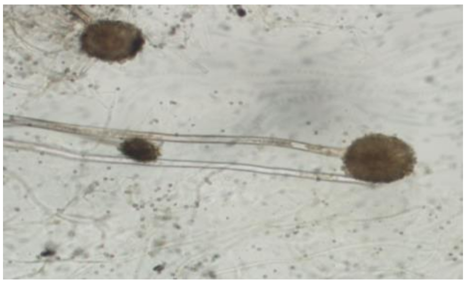<br>*A. niger MH079063.1*	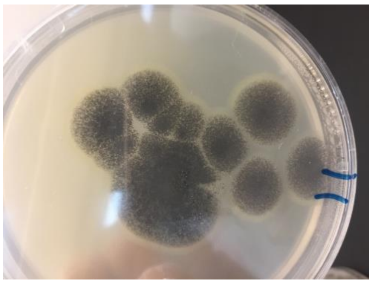<br>*A. niger MH079063.1*

Figure 4. (A) Under microscope (40X) and (B) on a petri dish photo of the 11 *Aspergillus sp.* Isolates producing lipase enzyme and examined in the current study.
