# Supplementary material for: Molecular, physiological, and biochemical characterization of extracellular lipase production by Aspergillus niger using submerged fermentation
Source: PeerJ. 2020 Jul 7;8:e9425. doi: 10.7717/peerj.9425 (PMC7350912; doi:10.7717/peerj.9425)
Supplement: Figure S5 — Amplicons were separated on agarose gels stained with ethidium bromide. Lane 1 through lane 11 are A. niger strains; MH111398.1, MH111399.1, MH057541.1, MH111400.1, MH078513.1, MH078565.1, MH111401.1, MH078566.1, MH078571.1, MH079049.1, MH079063.1 represent respectively. Lane 12 is 1-kb ladder (Quick-Load® 100 bp DNA Ladder, QIAGEN, Germany). [file peerj-08-9425-s005.pdf]

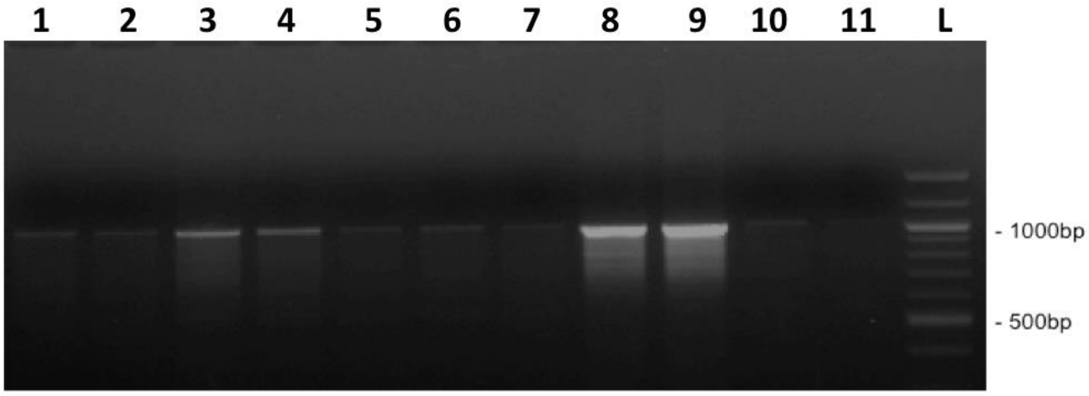

Figure 5. Lipase gene amplification of DNA extracted from the 11 *Aspergillus niger* strains. Amplicons were separated on agarose gels stained with ethidium bromide. Lane 1 through lane 11 are *A. niger* strains; MH111398.1, MH111399.1, MH057541.1, MH111400.1, MH078513.1, MH078565.1, MH111401.1, MH078566.1, MH078571.1, MH079049.1, MH079063.1 represent respectively. Lane 12 is 1-kb ladder (Quick-Load® 100 bp DNA Ladder, QIAGEN, Germany).
