# Supplementary material for: Molecular, physiological, and biochemical characterization of extracellular lipase production by Aspergillus niger using submerged fermentation
Source: PeerJ. 2020 Jul 7;8:e9425. doi: 10.7717/peerj.9425 (PMC7350912; doi:10.7717/peerj.9425)
Supplement: Table S1 [file peerj-08-9425-s006.pdf]

**Table 1.** Detection of (n=35) fungal isolates ability to hydrolyze olive oil using the phenol red medium

| Fungal isolates | Seed type / source | Halo of yellow ($cm^2$) |
|---|---|---|
| *Aspergillus sp.* | Cashew / Dammam | 48.36 |
| *Penicillium sp.* | Pistachio / Jubail | 47.73 |
| *Aspergillus sp.* | Pistachio / hassa | 45.84 |
| *Aspergillus sp.* | Bean / Dammam | 42.08 |
| *Aspergillus sp.* | Almonds / Jubail | 40.82 |
| *Aspergillus sp.* | Bean / Khobar | 40.19 |
| *Aspergillus sp.* | Cashew / Jubail | 40.19 |
| *Aspergillus sp.* | Pistachio / Jubail | 37.68 |
| *penicillium sp.* | Corn / Khobar | 35.8 |
| *Penicillium sp.* | Pistachio / Khobar | 34.54 |
| *Aspergillus sp.* | Almonds / Khobar | 34.54 |
| *Aspergillus sp.* | Pistachio / Dammam | 33.91 |
| *Penicillium sp.* | Cashew / Jubail | 30.14 |
| *Penicillium sp.* | Pistachio / Khobar | 29.52 |
| *Aspergillus sp.* | Pistachio / Hafr Al batin | 28.89 |
| *penicillium sp.* | Cashew / Jubail | 28.89 |
| *Aspergillus sp.* | Pistachio / Khobar | 28.26 |
| *Penicillium sp.* | Bean / Jubail | 27.63 |
| *Penicillium sp.* | Nut / Jubail | 26.38 |
| *Penicillium sp.* | Bean / Jubail | 21.98 |
| *Penicillium sp.* | Nut / Dammam | 21.98 |
| *Alternaria sp.* | Almonds / Hassa | 15.7 |
| *Aspergillus sp.* | Cashew / Jubail | - |
| *Aspergillus sp.* | Cashew / Jubail | - |
| *Fusarium sp.* | Corn / Dammam | - |
| *fusarium sp.* | Corn / Khobar | - |
| *fusarium sp.* | Corn / Jubail | - |
| *fusarium sp.* | Corn / Hassa | - |
| *fusarium sp.* | Corn / Hafr Al batin | - |
| *Alternaria sp.* | Pistachio / Dammam | - |
| *Alternaria sp.* | Pistachio / Khobar | - |
| *Alternaria sp.* | Pistachio / Hafr Al batin | - |
| *Alternaria sp.* | Bean / Dammam | - |
| *Alternaria sp.* | Almonds / Khobar | - |
| *Alternaria sp.* | Almonds / Hassa | - |
