# Supplementary material for: Molecular, physiological, and biochemical characterization of extracellular lipase production by Aspergillus niger using submerged fermentation
Source: PeerJ. 2020 Jul 7;8:e9425. doi: 10.7717/peerj.9425 (PMC7350912; doi:10.7717/peerj.9425)
Supplement: Table S2 [file peerj-08-9425-s007.pdf]

Table 2. The effect of range of temperatures on the enzymatic activity of the 5 highest lipase producers of *Aspergillus sp.* Isolates:

Temperature	Lipase activity (U/ml) ±S.D	Dry weight (g/flask) ±S.D	Diameter (cm) ±S.D
**15 ºC**			
A. niger MH111398	49.74 ± 2.35	0.708 ± 0.041	2.4 ± 0.1
A. niger MH111400	19.74 ± 4.44	0.442 ± 0.077	2.6 ± 0.1
A. niger MH078565	89.23 ± 21.54	0.438 ± 0.044	2.2 ± 0.1
A. niger MH078571	121.79 ± 16.18	0.572 ± 0.168	2.53 ± 0.058
A. niger MH079049	24.87 ± 8.92	0.42 ± 0.159	2.06 ± 0.058
**20 ºC**			
A. niger MH111398	155.64 ± 10.04	0.466 ±0.033	2.13 ± 0.153
A. niger MH111400	187.18 ± 6.89	0.365 ±0.123	2.1 ± 0.1
A. niger MH078565	214.87 ± 6.22	0.47 ± 0.085	2.26 ±0.252
A. niger MH078571	277.44 ± 4.51	0.50 ± 0.015	2.23 ±0.208
A. niger MH079049	247.95 ± 14.94	0.460 ± 0.125	2.2 ±0.1
**25 ºC**			
A. niger MH111398	324.3 ±3.87	0.41 ±0.058	5.4 ±0.625
A. niger MH111400	261.79 ±6.72	0.412 ±0.042	5.53 ±0.115
A. niger MH078565	276.41 ±5.12	0.291 ±0.023	5.5 ±0.1
A. niger MH078571	386.15 ±9.61	0.63 ±0.047	5.93 ±0.493
A. niger MH079049	402.31 ±3.53	0. ±0.026	4.77 ±0.153
**30 ºC**			
A. niger MH111398	334.36 ±6.72	0.323 ±0.099	5.1 ±0.1
A. niger MH111400	329.49 ±6.72	0.354 ±0.062	5.97 ±0.058
A. niger MH078565	407.436 ±8.58	0.306 ±0.164	5.23 ±0.252
A. niger MH078571	445.39 ±8.32	0.33 ±0.084	5.93 ±0.153
A. niger MH079049	445.9 ±6.45	0.34 ±0.057	5.5 ±0.1
**35 ºC**			
A. niger MH111398	409.49 ±3.47	0.29±0.047	4.4±0.794
A. niger MH111400	421.03 ±20.39	0.384 ±0.023	6.1 ±0.2
A. niger MH078565	436.92±3.35	0.3272 ±0.044	5.86 ±0.306
A. niger MH078571	487.44 ±13.08	0.375 ±0.082	4.3 ±0.608

A. niger MH079049	500.77 ±9.33	0.288 ±0.033	4.7 ±0.2
**40 ºC**			
A. niger MH111398.1	490.77 ±11.49	0.095 ±0.073	4.33 ±0.723
A. niger MH111400.1	470.51 ±11.95	0.225 ±0.055	3.73 ±0.153
A. niger MH078565.1	480.77 ±10.41	0.166 ±0.026	3.6 ±0.100
A. niger MH078571.1	575.9 ±9.56	0.292 ±0.030	3.6 ±0.794
A. niger MH079049.1	569.23 ±6.30	0.224 ±0.057	2.77±0.208
**45 ºC**			
A. niger MH078571.1	403.3 ±4.24	0.097 ±0.059	_
A. niger MH079049.1	524.87 ±6.94	0.090 ±0.090	_

* Results are averages of three replicates
