# Supplementary material for: Molecular, physiological, and biochemical characterization of extracellular lipase production by Aspergillus niger using submerged fermentation
Source: PeerJ. 2020 Jul 7;8:e9425. doi: 10.7717/peerj.9425 (PMC7350912; doi:10.7717/peerj.9425)
Supplement: Table S3 [file peerj-08-9425-s008.pdf]

**Table 3.** The effect of range of pH on the enzymatic activity of the 5 highest lipase producers of *Aspergillus sp.* Isolates:

| pH | Lipase activity (U/ml) ±S.D | Dry weight (g/flask) ±S.D | Diameter (cm) ±S.D |
|---|---|---|---|
| **3 pH** | | | |
| *A. niger MH111398* | 11.025±6.22 | 0.371±0.056 | _ |
| *A. niger MH111400* | 21.538±3.35 | 0.401±0.146 | _ |
| *A. niger MH078565* | 5.385±1.33 | 0.286±0.048 | _ |
| *A. niger MH078571* | 83.077±2.66 | 0.353±0.039 | _ |
| *A. niger MH079049* | 83.59±8.92 | 0.349±0.033 | _ |
| **3.5 pH** | | | |
| *A. niger MH111398* | 78.205±4.51 | 0.27±0.076 | _ |
| *A. niger MH111400* | 46.41±4.95 | 0.269±0.049 | _ |
| *A. niger MH078565* | 22.051±5.56 | 0.346 ±0.038 | _ |
| *A. niger MH078571* | 118.72±5.67 | 0.342±0.039 | _ |
| *A. niger MH079049* | 100±2.04 | 0.349±0.038 | _ |
| **4 pH** | | | |
| *A. niger MH111398* | 89.49±4.24 | 0.364±0.009 | _ |
| *A. niger MH111400* | 75.897±3.95 | 0.386±0.010 | _ |
| *A. niger MH078565* | 40.256±8.47 | 0.488±0.022 | _ |
| *A. niger MH078571* | 159.49±3.11 | 0.426±0.013 | _ |
| *A. niger MH079049* | 120.26±1.18 | 0.45±0.017 | _ |
| **4.5 pH** | | | |
| *A. niger MH111398* | 108.46±9.45 | 0.440±0.023 | 3.53±0.351 |
| *A. niger MH111400* | 102.05±7.15 | 0.428±0.007 | 3.43±0.058 |
| *A. niger MH078565* | 68.205±14.23 | 0.444±0.031 | 3.8±0.2 |
| *A. niger MH078571* | 183.59±2.22 | 0.485±0.013 | 3.93±0.306 |
| *A. niger MH079049* | 162.56±5.97 | 0.496±0.042 | 4.33±0.153 |
| **5 pH** | | | |
| *A. niger MH111398* | 158.72±8.30 | 0.24±0.104 | 4.27±0.252 |
| *A. niger MH111400* | 134.36±8.88 | 0.437±0.033 | 4.4±0.624 |
| *A. niger MH078565* | 93.08±5.38 | 0.392±0.025 | 5.067±0.208 |
| *A. niger MH078571* | 218.21±7.59 | 0.356±0.008 | 5.4±0.1 |
| *A. niger MH079049* | 205.64±3.20 | 0.382±0.017 | 5.53±0.208 |
| **5.5 pH** | | | |
| *A. niger MH111398* | 237.95±6.89 | 0.261±0.036 | 4.83±0.153 |
| *A. niger MH111400* | 161.54±4.07 | 0.332±0.036 | 4.47±0.153 |
| *A. niger MH078565* | 198.974±3.95 | 0.308±0.015 | 4.7±0.2 |

| | | | |
|---|---|---|---|
| *A. niger MH078571* | 278.97±5.12 | 0.312±0.065 | 4.73±0.252 |
| *A. niger MH079049* | 270.51±7.11 | 0.379±0.070 | 5.27±0.208 |
| **6 pH** | | | |
| *A. niger MH111398* | 348.72±5.00 | 0.477±0.042 | 4.3±0.265 |
| *A. niger MH111400* | 366.92±10.41 | 0.519±0.041 | 4.33±0.153 |
| *A. niger MH078565* | 335.39±12.71 | 0.514±0.009 | 4.57±0.208 |
| *A. niger MH078571* | 430±12.38 | 0.601±0.065 | 4.8±0.3 |
| *A. niger MH079049* | 423.85±12.09 | 0.591±0.074 | 4.37±0.321 |
| **6.5 pH** | | | |
| *A. niger MH111398* | 492.31±4.07 | 0.541±0.037 | 3.07±0.153 |
| *A. niger MH111400* | 470.77±2.04 | 0.584±0.022 | 3.67±0.208 |
| *A. niger MH078565* | 477.18±2.47 | 0.671±0.057 | 3.03±0.058 |
| *A. niger MH078571* | 577.44±2.47 | 0.638±0.048 | 3.6±0.1 |
| *A. niger MH079049* | 567.18±4.51 | 0.656±0.049 | 3.97±0.153 |
| **7 pH** | | | |
| *A. niger MH111398* | 503.85±8.87 | 0.643±0.051 | 4.37±0.153 |
| *A. niger MH111400* | 490.77±4.80 | 0.607±0.087 | 4.73±0.153 |
| *A. niger MH078565* | 497.44±11.13 | 0.638±0.046 | 4.67±0.208 |
| *A. niger MH078571* | 588.21±2.70 | 0.685±0.039 | 4.7±0.2 |
| *A. niger MH079049* | 592.56±5.56 | 0.686±0.024 | 4.63±0.058 |
| **7.5 pH** | | | |
| *A. niger MH111398* | 549.49±5.12 | 0.449±0.025 | 5.5±0.200 |
| *A. niger MH111400* | 510.26±2.35 | 0.276±0.024 | 5.27±0.153 |
| *A. niger MH078565* | 553.85±6.11 | 0.271±0.029 | 5.2±0.2 |
| *A. niger MH078571* | 610.77±5.81 | 0.27±0.027 | 5.6±0.1 |
| *A. niger MH079049* | 606.15±4.80 | 0.32±0.093 | 5.8±0.1 |
| **8 pH** | | | |
| *A. niger MH111398* | 484.1 ±2.91 | 0.401±0.074 | 4.8±0.265 |
| *A. niger MH111400* | 445.64±7.15 | 0.434±0.095 | 4.67±0.153 |
| *A. niger MH078565* | 477.95±8.47 | 0.346±0.059 | 4.6±0.1 |
| *A. niger MH078571* | 510±15.71 | 0.306±0.225 | 4.87±0.153 |
| *A. niger MH079049* | 487.18±1.18 | 0.512±0.047 | 5.3±0.252 |
| **8.5 pH** | | | |
| *A. niger MH078571* | 473.85±3.08 | 0.374±0.075 | 4.47±0.252 |
| *A. niger MH079049* | 470.51±8.23 | 0.475±0.064 | 4.7±0.2 |

* Results are averages of three replicates
