# Supplementary material for: Molecular, physiological, and biochemical characterization of extracellular lipase production by Aspergillus niger using submerged fermentation
Source: PeerJ. 2020 Jul 7;8:e9425. doi: 10.7717/peerj.9425 (PMC7350912; doi:10.7717/peerj.9425)
Supplement: Table S4 [file peerj-08-9425-s009.pdf]

Table 4. The effect of various incubation times on the enzymatic activity of the 5 highest lipase producers of *Aspergillus sp.* Isolates:

Incubation time	Lipase activity (U/ml)±S.D	Dry weight (g/flask) ±S.D	Diameter (cm) ±S.D
**3 Days**			
A. niger MH111398	585.39±14.41	0.656±0.028	5.4±0.361
A. niger MH111400	558.21±3.64	0.684±0.016	6.4±0.1
A. niger MH078565	575.39±12.38	0.527±0.027	6.1±0.173
A. niger MH078571	630.26±46.37	0.725±0.063	6.3±0.3
A. niger MH079049	633.85±17.74	0.728±0.076	6.3±0.289
**5 Days**			
A. niger MH111398	550±5.04	0.747±0.046	6.97±0.252
A. niger MH111400	508.97±5.24	0.797±0.018	7.33±0.153
A. niger MH078565	554.87±3.87	0.697±0.026	6.63±0.116
A. niger MH078571	608.97±2.70	0.836±0.052	7.27±0.322
A. niger MH079049	604.10±0.89	0.797±0.044	6.73±0.306
**7 Days**			
A. niger MH111398	423.33±12.69	0.899±0.047	7.07±0.306
A. niger MH111400	407.95±2.22	0.870±0.061	7.6±0.173
A. niger MH078565	503.85±7.69	0.829±0.043	6.77±0.252
A. niger MH078571	572.57±1.18	0.967±0.022	7.67±0.289
A. niger MH079049	554.36±4.51	0.915±0.034	7.43±0.153
**10 Days**			
A. niger MH111398	388.21±3.55	0.987±0.015	7.6±0.2
A. niger MH111400	361.28±4.24	0.999±0.028	7.67±0.153
A. niger MH078565	432.05±5.97	0.933±0.033	7.17±0.153
A. niger MH078571	552.82±4.37	1.05±0.084	7.83±0.153
A. niger MH079049	507.18±8.23	1.06±0.072	7.8±0.2
**15 Days**			
A. niger MH111398	348.21±1.17	1.133±0.186	7.83±0.153
A. niger MH111400	352.31±6.71	1.132±0.097	8±0.000
A. niger MH078565	367.69±42.01	0.989±0.014	7.97±0.058
A. niger MH078571	405.39±4.80	1.195±0.132	8±0.000
A. niger MH079049	385.39±2.04	1.246±0.054	8±0.000

* Results are averages of three replicates
