# Supplementary material for: Molecular, physiological, and biochemical characterization of extracellular lipase production by Aspergillus niger using submerged fermentation
Source: PeerJ. 2020 Jul 7;8:e9425. doi: 10.7717/peerj.9425 (PMC7350912; doi:10.7717/peerj.9425)
Supplement: Table S5 [file peerj-08-9425-s010.pdf]

Table 5. The effect of different carbon sources on the enzymatic activity of the 5 highest lipase producers of *Aspergillus sp.* Isolates:

Carbon source	Lipase activity (U/ml) ±S.D	Dry weight (g/flask) ±S.D	Diameter (cm) ±S.D
**Glucose**			
A. niger MH111398	608.21±0.44	0.825±0.130	3.23±0.208
A. niger MH111400	614.10±5.24	1.004±0.174	6.57±0.404
A. niger MH078565	608.21±1.60	0.925±0.121	5.8±0.625
A. niger MH078571	660.26±5.24	1.11±0.064	6.9±0.2
A. niger MH079049	647.18±5.24	1.245±0.095	7.17±0.153
**Fructose**			
A. niger MH111398	635.39±3.35	1.05±0.063	6.1±0.3
A. niger MH111400	625.9±4.44	1.24±0.063	6.2±0.265
A. niger MH078565	618.72±8.98	1.13±0.073	6.37±0.351
A. niger MH078571	717.44±20.89	1.39±0.082	6.5±0.2
A. niger MH079049	710.26±15.41	1.288±0.034	7.13±0.153
**Lactose**			
A. niger MH111398	608.21±0.44	0.904±0.122	6.67±0.153
A. niger MH111400	614.87±3.20	0.94±0.131	7.03±0.351
A. niger MH078565	607.44±0.44	0.941±0.075	6.3±0.265
A. niger MH078571	689.23±13.32	0.944±0.085	7.03±0.153
A. niger MH079049	670.51±17.14	0.957±0.209	7.03±0.252
**Galactose**			
A. niger MH111398	617.44±9.01	0.98±0.159	5.4±0.2
A. niger MH111400	610±0.77	1.029±0.160	6.73±0.306
A. niger MH078565	605.13±2.70	0.914±0.071	7.17±0.208
A. niger MH078571	609.49±0.89	0.985±0.016	7.37±0.252
A. niger MH079049	608.47±4.68	0.994±0.057	6.5±0.10
**Maltose**			
A. niger MH111398	648.72±7.15	0.561±0.012	5.37±0.115
A. niger MH111400	608.21±1.60	0.624±0.004	6.97±0.306
A. niger MH078565	611.28±0.44	0.555±0.040	7.23±0.208
A. niger MH078571	660±1.33	0.707±0.022	6.63±0.208
A. niger MH079049	615.64±4.64	0.695±0.036	5.37±0.208
**Sucrose**			
A. niger MH111398	551.03±8.98	0.674±0.073	3.53±0.153
A. niger MH111400	586.15±2.04	0.974±0.082	7.1±0.265
A. niger MH078565	599.23±1.33	0.917±0.018	3.83±0.153
A. niger MH078571	605.9±3.20	1.007±0.126	3.87±0.379
A. niger MH079049	601.54±4.28	1.109±0.116	3.17±0.351

**Starch**			
A. niger MH111398	554.87±3.87	0.4209±0.031	3.73±0.252
A. niger MH111400	562.82±4.95	0.462±0.035	5.93±0.252
A. niger MH078565	574.1±3.55	0.525±0.030	6.1±0.173
A. niger MH078571	592.56±4.51	0.612±0.027	5.07±0.404
A. niger MH079049	598.97±0.89	0.619±0.051	6.13±0.153

* Results are averages of three replicates
