# Supplementary material for: Molecular, physiological, and biochemical characterization of extracellular lipase production by Aspergillus niger using submerged fermentation
Source: PeerJ. 2020 Jul 7;8:e9425. doi: 10.7717/peerj.9425 (PMC7350912; doi:10.7717/peerj.9425)
Supplement: Table S6 [file peerj-08-9425-s011.pdf]

Table 6. The effect of different nitrogen sources on the enzymatic activity of the 5 highest lipase producers of *Aspergillus sp.* Isolates:

Nitrogen source	Lipase activity (U/ml) ±S.D	Dry weight (g/flask) ±S.D	Diameter (cm) ±S.D
**Peptone**			
A. niger MH111398	629.74±4.51	1.103±0.062	6.97±0.252
A. niger MH111400	620±8.87	1.308±0.037	7.07±0.208
A. niger MH078565	619.36±12.79	1.342±0.041	7±0.265
A. niger MH078571	713.33±2.22	1.396±0.059	7.6±0.200
A. niger MH079049	710.51±1.94	1.429±0.081	7.4±0.100
**Yeast extract**			
A. niger MH111398	669.49±10.61	1.037±0.038	5.57±0.153
A. niger MH111400	678.97±13.79	1.207±0.090	6.63±0.208
A. niger MH078565	708.97±5.12	1.133±0.066	6.7±0.100
A. niger MH078571	741.54±1.33	1.295±0.060	6.93±0.116
A. niger MH079049	734.36±3.87	1.309±0.068	7.27±0.208
**Beef extract**			
A. niger MH111398	508.72±8.85	0.979±0.079	3.47±0.058
A. niger MH111400	321.28±4.51	1.124±0.062	3.6±0.100
A. niger MH078565	502.82±3.47	1.04±0.086	4.97±0.058
A. niger MH078571	482.05±11.57	1.085±0.156	4.63±1.242
A. niger MH079049	522.05±3.95	1.204±0.111	6.53±0.153
**$NaNO_3$**			
A. niger MH111398	562.05±3.64	0.614±0.028	4.63±0.153
A. niger MH111400	561.28±25.76	0.702±0.071	3.93±0.116
A. niger MH078565	518.72±49.59	0.61±0.040	5.47±0.208
A. niger MH078571	433.59±3.64	0.729±0.036	4.8±0.200
A. niger MH079049	411.54±2.66	0.702±0.026	5.2±0.300
**Casein**			
A. niger MH111398	572.31±4.68	0.773±0.025	3.27±0.252
A. niger MH111400	448.21±8.92	0.911±0.026	3.3±0.265
A. niger MH078565	281.28±6.89	0.771±0.021	4.13±0.116
A. niger MH078571	315.64±4.24	0.892±0.064	4.47±0.153
A. niger MH079049	429.49±9.92	0.779±0.069	5.73±0.252
**KNO_3**			
A. niger MH111398	483.08±5.81	0.518±0.036	4.37±0.153
A. niger MH111400	438.21±5.40	0.607±0.037	3.83±0.153
A. niger MH078565	459.74±7.59	0.526±0.085	4.3±0.252

A. niger MH078571	486.67±7.11	0.819±0.070	3.6±0.100
A. niger MH079049	494.62±6.30	0.839±0.064	5.73±0.252

* Results are averages of three replicates
