# Supplementary material for: Molecular, physiological, and biochemical characterization of extracellular lipase production by Aspergillus niger using submerged fermentation
Source: PeerJ. 2020 Jul 7;8:e9425. doi: 10.7717/peerj.9425 (PMC7350912; doi:10.7717/peerj.9425)
Supplement: Table S7 [file peerj-08-9425-s012.pdf]

Table 7. The effect of various inoculum volume on the enzymatic activity of the 5 highest lipase producers of *Aspergillus sp.* Isolates:

Inoculum volume	Lipase activity (U/ml) ±S.D	dry weight (g/flask)
**0.5 ml**		
A. niger MH111398	235.10±36.40	0.208±0.059
A. niger MH111400	281.82±5.10	0.239±0.065
A. niger MH078565	293.49±42.47	0.183±0.034
A. niger MH078571	281.41±55.98	0.207±0.049
A. niger MH079049	301.1±67.69	0.255±0.062
**1 ml**		
A. niger MH111398	371.56±46.56	0.312±0.015
A. niger MH111400	438.56±57.62	0.397±0.017
A. niger MH078565	416.41±30.69	0.381±0.016
A. niger MH078571	467.08±7.13	0.459±0.040
A. niger MH079049	477.51±64.74	0.483±0.056
**1.5 ml**		
A. niger MH111398	470.62±21.19	0.685±0.094
A. niger MH111400	516.82±65.62	0.789±0.035
A. niger MH078565	462.69±24.23	0.645±0.039
A. niger MH078571	511.08±44.98	0.827±0.035
A. niger MH079049	549.33±49.39	0.845±0.096
**2 ml**		
A. niger MH111398	695.9±4.24	0.754±0.104
A. niger MH111400	710.51±3.11	0.824±0.042
A. niger MH078565	719.23±2.04	0.754±0.044
A. niger MH078571	767.18±3.47	0.848±0.083
A. niger MH079049	759.74±2.47	0.856±0.039
**2.5 ml**		
A. niger MH111398	709.74±3.64	0.824±0.044
A. niger MH111400	731.54±1.54	0.834±0.039
A. niger MH078565	735.64±1.94	0.879±0.016
A. niger MH078571	794.62±4.68	1.086±0.120
A. niger MH079049	787.69±2.04	1.018±0.059
**3 ml**		
A. niger MH111398	688.46±1.54	0.802±0.086

A. niger MH111400	691.28±2.47	0.819±0.060
A. niger MH078565	690±10.35	0.812±0.058
A. niger MH078571	757.95±4.70	1.065±0.108
A. niger MH079049	735.64±1.60	0.996±0.069

* Results are averages of three replicates
