# Supplementary material for: Molecular, physiological, and biochemical characterization of extracellular lipase production by Aspergillus niger using submerged fermentation
Source: PeerJ. 2020 Jul 7;8:e9425. doi: 10.7717/peerj.9425 (PMC7350912; doi:10.7717/peerj.9425)
Supplement: Table S8 [file peerj-08-9425-s013.pdf]

Table 8. The effect of various oil types on the enzymatic activity of the 5 highest lipase producers of *Aspergillus sp.* Isolates:

Oil type (%)	Lipase activity (U/ml) ±S.D	dry weight (g/flask) ±S.D	Diameter (cm) ±S.D
**Olive oil 1.5%**			
A. niger MH111398	557.69±4.07	0.864±0.136	5.2±0.436
A. niger MH111400	554.1±5.12	0.741±0.033	5.07±0.322
A. niger MH078565	586.41±0.44	0.623±0.030	4.3±0.458
A. niger MH078571	594.36±0.89	0.791±0.041	4.27±0.252
A. niger MH079049	571.8±3.47	0.703±0.072	4.43±0.116
**Olive oil 2%**			
A. niger MH111398	664.36±2.91	0.946±0.093	5.77±0.306
A. niger MH111400	670.51±6.17	1.014±0.188	4.97±0.451
A. niger MH078565	697.69±5.04	0.955±0.040	4.37±0.208
A. niger MH078571	735.9±1.78	1.137±0.149	3.27±0.252
A. niger MH079049	728.21±4.24	1.232±0.142	4.17±0.416
**Olive oil 2.5%**			
A. niger MH111398	664.87±13.26	1.072±0.103	2.6±0.322
A. niger MH111400	598.21±6.54	1.034±0.155	2.7±0.751
A. niger MH078565	672.05±21.54	1.277±0.149	4.27±0.404
A. niger MH078571	674.36±1.18	1.041±0.110	2.53±0.058
A. niger MH079049	688.21±0.44	1.039±0.093	3.9±0.529
**Corn oil 1.5%**			
A. niger MH111398	507.95±65.54	0.617±0.059	4.23±0.208
A. niger MH111400	477.95±17.45	0.52±0.086	6.63±0.322
A. niger MH078565	484.1±15.04	0.717±0.029	6.17±0.208
A. niger MH078571	450.77±29.80	0.836±0.050	4.17±0.153
A. niger MH079049	481.54±24.24	0.848±0.126	4.9±0.265
**Corn oil 2%**			
A. niger MH111398	520.26±2.47	0.613±0.031	3.77±0.252
A. niger MH111400	537.44±6.72	0.663±0.085	4.73±0.252
A. niger MH078565	525.13±21.88	0.719±0.091	3.57±0.208
A. niger MH078571	543.08±40.09	0.863±0.112	4.7±0.361
A. niger MH079049	566.67±94.95	0.834±0.100	4.77±0.306
**Corn oil 2.5%**			
A. niger MH111398	651.03±8.23	0.662±0.054	4.5±0.2
A. niger MH111400	645.64±5.67	0.614±0.102	3.87±0.306
A. niger MH078565	608.46±1.54	0.620±0.079	3.8±0.300

A. niger MH078571	618.97±14.72	0.838±0.078	4.17±0.252
A. niger MH079049	590.51±31.76	0.888±0.112	4.3±0.2
**Sun flower 1.5%**			
A. niger MH111398	534.87±47.78	0.849±0.056	4.07±0.306
A. niger MH111400	517.69±1.18	0.822±0.038	6.6±0.361
A. niger MH078565	484.36±0.44	0.692±0.064	6.23±0.208
A. niger MH078571	532.31±18.46	0.703±0.056	5.03±0.351
A. niger MH079049	540.51±25.38	0.777±0.023	5.9±0.173
**Sun flower 2%**			
A. niger MH111398	540.77±2.77	0.908±0.160	6.87±0.153
A. niger MH111400	540±51.61	0.844±0.058	6.5±0.500
A. niger MH078565	536.41±2.35	0.739±0.067	4.57±0.058
A. niger MH078571	537.95±5.88	0.88±0.048	4.77±0.208
A. niger MH079049	546.16±1.54	0.88±0.016	5.6±0.100
**Sun flower 2.5 %**			
A. niger MH111398	538.97±2.35	0.775±0.061	4.17±1.012
A. niger MH111400	517.69±15.13	0.745±0.037	4.7±0.300
A. niger MH078565	498.72±40.77	0.836±0.120	5.27±0.252
A. niger MH078571	532.31±6.92	0.681±0.061	4.87±0.153
A. niger MH079049	540.53±54.23	0.692±0.079	4.6±0.100
**Castor oil 1.5%**			
A. niger MH111398	473.08±9.36	0.685±0.045	4.5±0.173
A. niger MH111400	488.46±4.68	0.924±0.238	3.87±0.231
A. niger MH078565	507.44±15.10	0.845±0.085	3.23±0.252
A. niger MH078571	497.69±8.74	0.807±0.014	4.37±0.252
A. niger MH079049	502.31±3.53	0.81±0.047	5.17±0.153
**Castor oil 2%**			
A. niger MH111398	505.13±5.97	0.766±0.046	3.43±0.208
A. niger MH111400	577.18±7.47	0.795±0.062	3.2±0.2
A. niger MH078565	628.46±18.95	0.764±0.161	2.97±0.058
A. niger MH078571	694.36±25.19	0.779±0.074	4.17±0.153
A. niger MH079049	698.72±7.34	0.934±0.036	5.43±0.116
**Castor oil 2.5%**			
A. niger MH111398	491.54±7.34	0.875±0.019	2.1±0.1
A. niger MH111400	572.05±13.20	0.847±0.173	1.97±0.153
A. niger MH078565	565.39±2.77	1.04±0.296	3.07±0.493
A. niger MH078571	565.9±5.12	0.926±0.102	3.23±0.252

A. niger MH079049	588.46±2.04	0.929±0.160	5.3±0.3
**Palm oil 15%**			
A. niger MH111398	662.82±5.24	0.657±0.053	3.17±0.153
A. niger MH111400	667.69±14.68	0.605±0.034	3.03±0.058
A. niger MH078565	710.51±5.77	0.676±0.086	2.47±0.058
A. niger MH078571	735.64±1.60	0.743±0.136	3.7 3±0.252
A. niger MH079049	731.8±0.44	0.582±0.057	5.03±0.252
**Palm oil 2%**			
A. niger MH111398	692.56±3.11	0.677±0.059	3.07±0.153
A. niger MH111400	710.26±11.26	0.645±0.125	4.1±0.1
A. niger MH078565	726.41±3.64	0.645±0.054	3.53±0.058
A. niger MH078571	772.05±0.44	0.656±0.031	4.97±0.208
A. niger MH079049	758.46±18.80	0.641±0.067	5.07±0.306
**Palm oil 2.5%**			
A. niger MH111398	663.85±2.04	0.639±0.001	2.93±0.116
A. niger MH111400	667.44±8.23	0.638±0.048	3.97±0.153
A. niger MH078565	635.39±9.61	0.625±0.094	5.37±0.379
A. niger MH078571	740.77±2.04	0.642±0.070	4.07±0.116
A. niger MH079049	723.078±8.14	0.666±0.042	4.9±0.1
**Soy bean 1.5%**			
A. niger MH111398	535.13±7.74	0.631±0.103	3.8±0.200
A. niger MH111400	540.52±2.35	0.597±0.076	5.63±0.153
A. niger MH078565	508.72±14.29	0.669±0.005	3.8±0.200
A. niger MH078571	530.52±30.19	0.661±0.053	4.7±0.200
A. niger MH079049	538.47±4.28	0.69±0.023	4.67±0.116
**Soy bean 2%**			
A. niger MH111398	550.26±2.91	0.603±0.025	4.17±0.153
A. niger MH111400	557.69±10.91	0.721±0.044	4.5±0.100
A. niger MH078565	547.95±2.47	0.715±0.033	4.07±0.208
A. niger MH078571	535.9±4.44	0.675±0.048	5.37±0.252
A. niger MH079049	558.72±3.64	0.620±0.074	5.73±0.252
**Soy bean 2.5%**			
A. niger MH111398	537.7±7.05	0.712±0.011	4.13±0.153
A. niger MH111400	524.11±2.70	0.698±0.030	6.03±0.252
A. niger MH078565	519.75±8.47	0.686±0.066	4.27±0.153
A. niger MH078571	522.57±5.12	0.643±0.011	5.43±0.379
A. niger MH079049	525.39±4.07	0.705±0.067	5.3±0.173

* Results are averages of three replicates
